# The neurosociological paradigm of the metaverse

**DOI:** 10.3389/fpsyg.2024.1371876

**Published:** 2025-01-07

**Authors:** Olga Maslova, Natalia Shusharina, Vasiliy Pyatin

**Affiliations:** ^1^Department of Science, Eurasian Technological University, Almaty, Kazakhstan; ^2^Baltic Center for Neurotechnologies and Artificial Intelligence, Immanuel Kant Baltic Federal University, Kaliningrad, Russia; ^3^Neurointerfaces and Neurotechnologies Laboratory, Neurosciences Research Institute, Samara State Medical University, Samara, Russia

**Keywords:** metaverse, neurosociological paradigm, social brain networks, hyperscanning, interbrain synchrony, social virtual reality, ethics, neurodiversity

## Abstract

Metaverse integrates people into the virtual world, and challenges depend on advances in human, technological, and procedural dimensions. Until now, solutions to these challenges have not involved extensive neurosociological research. The study explores the pioneering neurosociological paradigm in metaverse, emphasizing its potential to revolutionize our understanding of social interactions through advanced methodologies such as hyperscanning and interbrain synchrony. This convergence presents unprecedented opportunities for neurotypical and neurodivergent individuals due to technology personalization. Traditional face-to-face, interbrain coupling, and metaverse interactions are empirically substantiated. Biomarkers of social interaction as feedback between social brain networks and metaverse is presented. The innovative contribution of findings to the broader literature on metaverse and neurosociology is substantiated. This article also discusses the ethical aspects of integrating the neurosociological paradigm into the metaverse.

## 1 Introduction

### 1.1 From sociology to neurosociology

Social interaction, as a key social behavioral motive in human society, is the subject of sociology (Suckert, 2022; Beckert and Suckert, 2021; Abbott, 2016). Sociology uses qualitative and quantitative social research methods to study the social experiences of small and large groups of people (Foster et al., 2021). Until recently, sociology has lacked a direction related to the study of social behavioral acts in the paradigm of neural mechanisms of social homeostasis with the participation of social brain centers (Matthews and Tye, 2019). Studies of socially motivated behavior that is regulated by the main social neural networks of the human brain are beginning to appear in the literature. These social neural networks are the central executive network (CEN), the salience network (SN)—which specializes in the control of social behavior—the default mode network (DMN), and the subcortical network (SCN) (Feng et al., 2021).

Previously, numerous functional magnetic resonance imaging (fMRI) studies have substantiated the role of the social neural networks of the brain in the context of various forms of socially determined responses in the static “brain-tasks” conditions (Montague et al., 2002). However, as part of the evolution of new knowledge about the social neural networks and pioneering work in the field of fMRI-based hyperscanning (Montague et al., 2002), the concept of neurosociology was formed (TenHouten et al., 2022). It has become evident that the development of the subjective nature of social experience, as a key element of its integration into social behavior, is a function of the neural networks of the social brain. At the same time, significant social experience is formed by the social neural networks during the process of dynamic face-to-face communication (Hirsch et al., 2021; Schwartz et al., 2024), which can be studied and analyzed in real time from the standpoint of neurosociology. It should be emphasized that the “gold standard” of human communication (face-to-face), which represents the majority of the people’s daily experience, especially between close people (Subrahmanyam et al., 2020), has never been a subject of interest for sociologists. The studies by many authors prove that this is the only and most important form of interaction at all stages of human age-related social development (Davies, 2012; Aslan et al., 2024; de Leeuw et al., 2024), as it offers a vibrant form of social interaction, with its synchronous exchange of multimodal social signals.

As digital spaces such as the metaverse emerge, the need to understand social interactions through the lens of neurosociology becomes increasingly critical. The metaverse offers a unique environment where neurosociological principles can be applied to study social behavior in ways the traditional settings cannot. Indeed, neurosociology is focused as much as possible on uncovering the neurophysiological mechanisms of a key form of “face-to-face” social interaction between people (de Leeuw et al., 2024), which has been impossible to do within sociological research. In modern conditions, when the Internet is being replaced by the metaverse with its cardinal feature of “the feeling of being fully present,” neurosociology under conditions of dynamic hyperscanning becomes a powerful scientific and practical direction in studying and reconstructing the new digital subjective and objective world. The metaverse has great potential to revolutionize our understanding of social interactions by leveraging advanced methodologies, such as hyperscanning and interbrain synchrony. In addition, the merging of neurosociology and the metaverse presents unprecedented opportunities for both neurotypical and neurodivergent individuals.

### 1.2 The neurosociological paradigm and neurodivergence

Indeed, the neurosociological paradigm, in alliance with the metaverse opens unlimited perspectives of social interactions for all people. Despite that, majority of the people are “neurotypical” (Alkhaldi et al., 2021). This means that the social brain of “neurotypical” people, and the associated main social neural networks (CEN, DMN, and SN), operate and process information in the way that society expects, meaning “neurotypical” people fit into the norm of thinking (Szechy and O’Donnell, 2024). At the same time, one one-fifth of people on the planet are considered neurodivergent (Goldberg, 2023). Neurodiversity is characterized by diversity of thought and social dynamics, which are natural, healthy, and valuable forms of human diversity because there is no naturally “normal” or “right” style of the human mind. Neurodivergent states are not a deviation from the norm, but natural differences in the functioning of the human brain that do not need to be “fixed” or “corrected” (Milton, 2012; Milton et al., 2022). Meanwhile, neurodivergent individuals show unique manifestations in the cognitive domains, such as verbal learning, planning, attention, emotional processing, and memory functions (Mohamed et al., 2021; Velikonja et al., 2019). However, neurodivergent children, adolescents, adults, and their families with neurodiversity sometimes face significant barriers to accessing services and society. For example, the well-known dual empathy problem (Milton, 2012; Milton et al., 2022; Crompton et al., 2021) is associated with the great difficulty of neurodivergent individuals to empathize with each other.

In contrast to the medical and social models of neurodivergence (Casanova and Widman, 2021; Dwyera, 2022; Moore et al., 2024), the benefits of integrating the metaverse with neurosociology technologies have enormous potential for personalized solutions to the specific problems people with a whole range of different neurodivergent conditions face currently.

So, the metaverse, equipped with neurosociological tools such as hyperscanning, offers a new frontier for addressing the dual empathy problem in neurodivergent populations. It is important to emphasize that the metaverse can equally provide a rich digital social form of behavior for neurotypical and neurodivergent individuals (Hutson, 2022), given the uniqueness of the methodology of the neurosociological paradigm in the metaverse.

### 1.3 Methodological basis of the neurosociological paradigm in the metaverse

The methodological basis for realizing the unique capabilities of the neurosociological paradigm in the metaverse is the hyperscanning technology and the phenomenon of synchrony of neurophysiological brain biomarkers during real social interaction. As it is known, initially and until now the brain hyperscanning started to be applied in the study of social interaction in the dyads paradigm, when neurosynchrony of electrical (electroencephalography [EEG] method) and/or metabolic activity (fNIRS method) of the social neural networks is recorded and analyzed, as a rule, in two subjects under the dynamic conditions of “face-to-face” communication (Dikker et al., 2017; Liu et al., 2017; Wang et al., 2018; Barde et al., 2020; Feng et al., 2021; Hirsch et al., 2021; Schwartz et al., 2024).

Registration and real-time analysis of interbrain neural synchrony in the cortex cerebral hemispheres objectively indicate the success of targeted social understanding and real opportunities for empathy formation depending on the context of social activity in the virtual environment. Obviously, the methodology of the neurosociological paradigm in the metaverse can be supplemented by the phenomenon of synchronization taking place during the social interaction at the level of physiological parameters of the executive functions. For example, the synchronized physiological responses have been shown to be recorded in dyads such as the electrodermal activity and the peripheral skin temperature (Hanshans et al., 2024), the heart rate variability (HRV), and pulse variability (Rockstroh et al., 2019; Immanuela et al., 2023). The stronger HRV synchrony during conflict in pairs can predict greater mood reactivity (Wilson et al., 2018). Physiological synchrony is explained by the specific emotional state of participants in virtual reality (VR). The VR technologies have potential as stress reduction techniques (Ladakis et al., 2024), and personalized VR experience increases emotional empathy (Martingano et al., 2021). The phenomenology of brain-to-brain coupling synchrony and synchrony of physiological system’ parameters support the view that the social virtual environments create a “sense of total presence” (Hameed and Perkis, 2024; Moharana et al., 2023) and identity (Yang et al., 2024a,b) with the real environment. Methodological perspectives in the neurosociological paradigm of the metaverse are open for revolutionary development, as the peculiarity of VR is that it needs to be richer and personalized to develop social communication (Kandalaft et al., 2013; Didehbani et al., 2016; Yin et al., 2022; Liu et al., 2024).

So, our general point of view is that in the metaverse, hyperscanning can be used to monitor interbrain synchrony during virtual meetings, providing insights into group dynamics and decision-making processes that are not possible in traditional settings. The application of hyperscanning methodology in the communication process of subjects immersed in the metaverse provides technological novelty, which has human adoption as one of its first and the most important challenges (dos Santos et al., 2024).

### 1.4 Theoretical basis of the neurosociological paradigm in the metaverse

The theoretical basis for realizing the methodological perspective of the neurosociological paradigm in the metaverse is the symbolic interactionism theory (Lee and Joo, 2022) and Piaget’s theory of genetic epistemology (Ke and Qiwei, 2020).

According to the symbolic interactionist theory, the meaning, interaction, and human activity are placed at the center of understanding social life (Sandstrom and Kleinman, 2005)—the neurophysiological content of which is the subject of neurosociology. The symbolic interactionism theory analyzes the way society is created and maintained through the personal, repeated, and meaningful interactions between people (collective behavior and social movement), as well as social context and environment (Carter and Fuller, 2016). Moreover, the notions of symbolic signals and improvised self-representations in symbolic interactionism have a wide application to the study of computer-mediated communication and self-construction in social environments (Udoudom et al., 2024). The importance of the evolution of the neurosociological paradigm within the symbolic interactionist theory is supported by the key role of the universal seamless cross-xR experience in the metaverse (Tümler et al., 2022), which is created by the combination of VR with a personal computer and Hololens 2, in which the human dimension most fully realizes people’s abilities to understand and use the data shared, as well as their willingness to collaborate (Yang et al., 2024a,b). Another descriptive aspect of the neurosociological paradigm within the symbolic interactionist theory stems from the key role of dynamically changing symbols of VR in real time (Shen et al., 2022), which are measured by the neurosociological hyperscanning technology. This is confirmed by a recent study in the metaverse paradigm based on the symbolic interactionism theory (Lee and Joo, 2022). The authors found that personal, repetitive, meaningful interactions between individuals in the real world shape the users’ own identities and differences in the metaverse, which subsequent analysis of the data showed they related to the presence or absence of ongoing communication with others in the real world (Lee and Joo, 2022).

Another crucial theoretical basis for the neurosociological paradigm in the metaverse is Piaget’s theory of genetic epistemology, as it implies the adaptation of the organism to the environment and solution of “hard problems,” such as the “problem of the other mind” (Ke and Qiwei, 2020). In our subject area, the first aspect is related to the adaptation of the social brain to the social interaction processes in VR, and the second is to the adaptation of the social cognitive state under neurodivergent conditions. Yin et al. (2022) proposed a social virtual model of restructuring the patients’ schema modes in personality disorders, which can be achieved due to the VR exceptional ability to create a “sense of total presence” (Slater and Sanchez-Vives, 2016; Riches et al., 2019; Hameed and Perkis, 2024) and identity (Yang et al., 2024a,b).

Piaget’s theory of genetic epistemology, when applied within the metaverse, could provide a framework for understanding how users adapt to and learn from immersive digital environments, thereby reshaping social norms and behaviors. The social virtual model created by the authors for patients with personality disorders is ideally suited to lead in its clinical application to adaptive and more inclusive patient interactions with the real world to support adaptive behavior and restore a capability of emotional regulation. In contrast to the clinically oriented model (Yin et al., 2022) the neurodivergent model (Dwyera, 2022), within the neurosociological paradigm in the metaverse according to Piaget’s theory of genetic epistemology will aim to improve social communication based on neurophysiological mechanisms of neuroplastic remodeling of the social neural networks.

### 1.5 Development of the neurosociological paradigm in the metaverse

**Figure 1:**
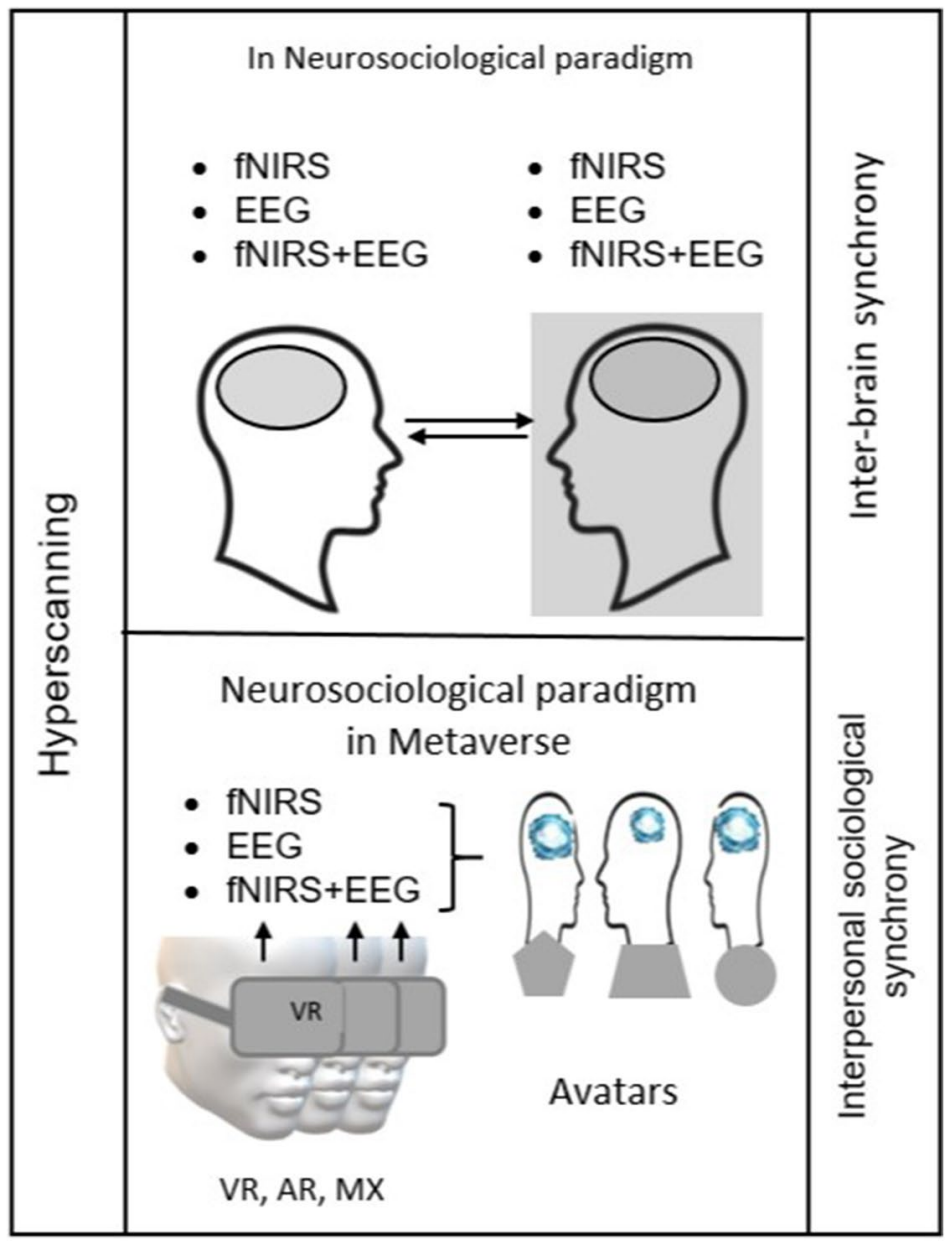
Brain-to-brain hyperscanning using fNIRS and EEG as the key methods for studying social interaction synchrony within the neurosociological paradigm. The figure demonstrates how different conditions (free, inpatient, and laboratory) enable the detailed mapping and synchronization of the brain signals, which is essential for understanding social behavior in both real and virtual environments (above). Neurosociological methodology of multipersonal hyperscanning and interbrain synchrony via controlled avatars and digital virtual objects of the social virtual platforms are necessary for revolutionizing our understanding of social interactions by leveraging advanced methodologies (below).

The development of the neurosociological paradigm in the metaverse can be viewed as a three-phase process: initial integration of neurosociological tools, real-time monitoring and feedback, and eventual adaptation of social norms within the virtual environments. In its most conceptual form, it can be stated that the theory, methodology, and software of the neurosociological paradigm are already represented in objective reality (Figure 1).

Currently, the participants wearing fNIRS or EEG head helmets can enter the virtual reality of different content via VR, augmented reality (AR), or mixed reality (MR). Our study demonstrates how neurosociological tools are being integrated with VR technologies. Thus, in the comparative study on interbrain synchrony in virtual environments using hyperscanning, an increase in interbrain synchrony during collaborative tasks in VR is shown (Gumilar et al., 2021). Moreover, intra- and interbrain connections have patterns of neural synchronization across all EEG bands in both leaders and followers (Chuang et al., 2024). However, the studies were conducted outside the metaverse paradigm in these studies.

The virtual world (metaverse) is represented by digital twins, virtual elements that may or may not correspond to the compartments of the physical world and, more importantly, interact with the physical world. Moreover, the metaverse will be fully realized if the interaction between the two worlds has the highest level of independence (Shi et al., 2023; AbuKhousa et al., 2023). The main events taking place in the metaverse are generated by people who interact with each other, for example, through the avatars (Rogers et al., 2022; Crespo-Pereira et al., 2023), transact, create, play, work, and teleport through spaces (AbuKhousa et al., 2023). The evolution of the metaverse is associated with the development of digital technologies (fifth-generation [5G] networks, blockchain, artificial intelligence (AI), Internet of Things, computing, VR, AR, MR, and extended reality). The social interactions of users in the metaverse digital space by the avatars facilitate the joint creation of virtual values that, translationally with the participation of AI, can determine the technological progress of the physical universe (Buhalis et al., 2023). Moreover, social interaction during a three-dimensional (3D) immersive experience leads to a multicognitive lifestyle (Buhalis et al., 2023). The majority of the studies of the metaverse phenomenon are viewed from a technological perspective. However, it should be emphasized in future studies that the metaverse has an essential social and neurosocial component (Park and Kim, 2022). This is because people’s social interactions in the physical world are determined by the function of the social brain’s neural networks (Adolphs, 2009), fostering the users’ immersion in the metaverse platforms.

The stage of the real-time monitoring and feedback between the neurosociological tools and the metaverse (Figure 1) can be revolutionized by targeting the neurosociological paradigm in the metaverse to study the main social neural networks—the default mode networks (DMN), the salience network (SN), the central executive network (CEN), and the subcortex network (SCN) of rewards (Feng et al., 2021) represented by striatum in the context of social behavior. The focus of the neurosociological paradigm in the metaverse on the CEN will make it possible to reveal new aspects of its global role in controlling all main neural networks of the brain and especially social neural networks. Based on this, it can be assumed that socially motivated activity in the metaverse will be aimed at stimulating the processes of the neural network’s plasticity in the social brain, which can be monitored and analyzed by the hyperscanning technology in real time. This point of view is confirmed by the significant increase in intra- and internetwork integration of SN and CEN during a video game, according to fMRI data preprocessing in professionals relative to amateurs (Gong et al., 2016). In the same aspect, the brain-to-brain hyperscanning based on fNIRS and EEG methods is an alternative and promising method for social brain research in neurosociology (Hirsch et al., 2021; Hirsch et al., 2022; Maslova et al., 2022). The combination of EEG and fNIRS techniques provides spatial and temporal resolution of the activity of the same brain point, which promotes investigating the neurovascular interaction due to recruited neuronal activity (Lin et al., 2023; Blanco et al., 2024). Brain-to-brain hyperscanning (EEG and/or fNIRS)—synchronization of neural activity in the transcortical context—is a correlate of positive social interaction between subjects (Melloni et al., 2007; Maslova et al., 2022) and can reach the revolutionizing level in the metaverse. The transforming theoretical basis of the real-time monitoring and feedback stage is the theory of quantization of social interaction in the metaverse based on the neurosociological paradigm we propose, which will be discussed in the “Discussion” section.

The highest form of development of the neurosociological paradigm in the metaverse is the adaptation of social norms within the virtual environments, set by the social value of the metaverse, which is the natural digital space where the human social brain will operate. Therefore, the neurosociological perspective enables the creation of meaningful and impactful experiences and a better understanding of social, cognitive, and emotional processes that drive human behavior in the metaverse (Crespo-Pereira et al., 2023). The future of the metaverse is related technological innovation and its acceptance by society in the form of adaptation of social norms in the virtual environment of the metaverse. The enormous neurosociological dataset in the metaverse by the real-time monitoring and feedback will be exploited through the implementation of blockchain (Elsadig et al., 2024) and AI (Soliman et al., 2024), the application of which is already widely analyzed in neuroscience (Harris, 2024), but a detailed analysis of their role in the future development of the neurosociological paradigm in the metaverse is beyond the scope of this study.

As a general conclusion, we can say that this study aims is to uncover the transformative potential of integrating the neurosociological paradigm with the metaverse for the present and the future of humanity’s digital world.

## 2 Social brain as a neurosociological transformation of sociology

The initial impetus for the transformative potential of modern integration of neurosociology with the metaverse was due to the advances in neuroscience, especially brain neuroimaging in different social contexts, which showed that the relationship between mind and society is a function of neural networks of the social brain (Adolphs, 2009; Franks, 2010; Franks and Turner, 2013; Kalkhoff et al., 2016; Firat, 2019). Over the past two decades, the lines of research on neural networks mediating human social interactions have emerged in neuropsychology (Lin et al., 2012; Barrett and Satpute, 2013), social neuroscience (Adolphs, 2009; Cacioppo and Decety, 2011), neuroeconomics (Walter et al., 2005), and evolutionary neuroscience (Chin et al., 2023). Studies in evolutionary neurobiology have shown a unique organization of neural networks in the human brain, namely, the transmission of information through the parallel pathways that act as the main links between unimodal and transmodal neurosystems (Griffa et al., 2023). This evolution of communication development in the human brain differs significantly from that in the primate brain. It determines the peculiarities of its higher social functions and, consequently, approaches to their research “neurosupport.” This has been achieved by applying comparative neuroimaging to investigate the structural and functional specialization of neural networks from an evolutionary perspective (van den Heuvel et al., 2016; Friedrich et al., 2021).

The personal, repeated, meaningful interactions between people and dynamic human activity are the core of understanding social life (Sandstrom and Kleinman, 2005; Carter and Fuller, 2016). Based on the symbolic interactionism theory (Lee and Joo, 2022), in the metaverse this understanding is achieved through the neurosociological paradigm analyzing social dynamically changing the VR symbol signals in real time (Shen et al., 2022), the universal seamless cross-xR experience in the metaverse (Tümler et al., 2022) and improvised self-representations which have a wide application to the study of computer-mediated communication and self-construction in social environments (Udoudom et al., 2024). All these symbolic signals are measured by the neurosociological hyperscanning technology and synchrony in the social neural networks as the compartments of the social brain. When applied within the metaverse, Piaget’s theory of genetic epistemology could provide a framework for understanding how users adapt to and learn from immersive digital environments, which is also due to the flexible processes of the neural networks of the social brain.

The social brain conditions all forms of human social interaction, when there is a reciprocal relationship between two or more actors (Feng et al., 2022). Sociology, as a science that studies social interaction outside the concept of the social brain, looks to the present and the past, and still, to a large extent and up to the present, “looks back at the causes funneling into a final result” (Beckert and Suckert, 2021; Abbott, 2016). At the same time, some sociologists draw attention to the fact that the new paradigm in sociology, according to which the most significant theories of sociology are primarily concerned with the future, is promising (Suckert, 2022). The prospect of successfully addressing the sociology’s most significant theories can be related to the metaverse, equipped with neurosociological tools such as hyperscanning, and offering a new frontier for addressing the social brain. Especially since the modeling of social exchange shows that productive forms generate the strongest micro-order (Lawler et al., 2008). This position is significant for neurosociology because it reveals the brain mechanisms of such social behavior in the metaverse modeling. One of the key directions of transformation “back to the future” (Suckert, 2022) is precisely the relationship between neurosociology and the metaverse. Neurosociological paradigm in the metaverse in the present/past and the imagined future, opens a new approach to solving the issues of sociological research. Moreover, according to the literature, many sociological publications are rediscovering the future as a theoretical perspective, analytical category and object of study (Rona-Tas, 2020; Beckert and Suckert, 2021).

Current basic understanding of the key role of the social brain in human social cognition have been formed by studying such socially related functions of the neural networks as the mirror system, and the four specific brain regions considered to have a role in social cognition: the posterior superior temporal sulcus and the adjacent temporoparietal junction; the amygdala; the temporal poles; the medial prefrontal cortex; and the adjacent anterior cingulate cortex (ACC) (Frith, 2007; Frith and Frith, 2010; Adolphs, 2009). These neural network compartments in the social brain paradigm are a part of the structure of the main social neural networks of the human brain, DMN, SN, and CEN, which determine different forms of social behavior, and, also, according to Feng et al. (2021), subcortical network, as a striatum, a social rewarding network.

In the framework of the symbolic interactionism theory (Lee and Joo, 2022), the socially relevant symbolic factors and thus influence social connectedness and social well-being of individuals modulate activity in the medial prefrontal cortex and ACC regions as a CEN compartment (Kim and Sul, 2023). On the contrary, with the long environmental monotony and prolonged physical and social isolation, there is a structural and functional reorganization of the main neural network in the form of lower gray-matter volume in the right dorsolateral prefrontal cortex, the left orbitofrontal cortex, and the left parahippocampal gyrus than in the controls (Stahn et al., 2019), and disturbed sleep, impaired cognitive abilities, negative emotions, interpersonal tension, and conflicts (Palinkas and Suedfeld, 2008). According to the symbolic interactionism theory, any change in the goal of social behavior can transform the social specificity of intraneural network interactions (Lockwood et al., 2020) and be a causal factor affecting social outcomes (Suckert, 2022), which diversely can be modeled in the social environment of the metaverse. The reward responses that shape human behavior (Bhanji and Delgado, 2014) and show that the neural circuitry of reward, particularly the striatum, is also involved in processing social information and making decisions in social situations, which allows understanding the development of social experience, social interaction, motivation, and decision-making. Consequently, the compartments of the social brain along the ventromedial prefrontal cortex, lateral prefrontal cortex, the amygdala, and striatum play roles in social behavior (Cardinal et al., 2002).

Integrating social brain research within the framework of Piaget’s theory of genetic epistemology provides a better understanding of the correlates of neuroplasticity’s restructuring of the complex hierarchy in the networks of the social brain—DMN, SN, and CEN—in different types of social interactions.

**Figure 2:**
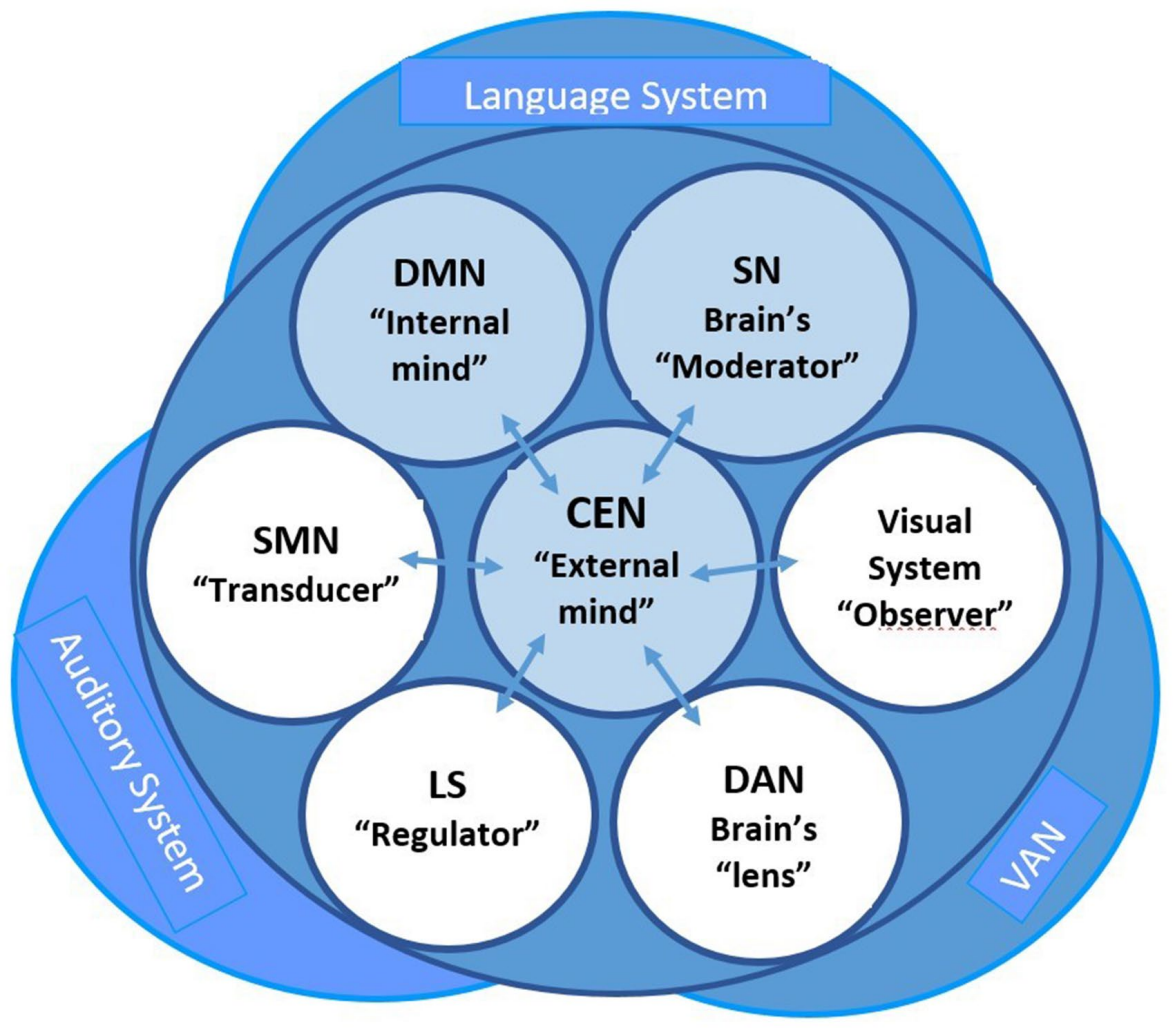
Interaction of the main neural networks in social behavior, highlighting the role of CEN, DMN, and SN in modulating cognitive and emotional processes. This balance is critical for optimizing social interactions within the dynamic environments of the metaverse. In doing so, multistage quanta of the neurosociological paradigm in the metaverse increase the information processing at the global internetwork and local intranetwork levels of DMN and CEN in their current control by SN.

In the human brain, variability in functional connectivity is highest in the frontal, temporal, and parietal regions of the association cortex, which with their functions of sensory awareness, visual imagination, speech, and spatial learning, perform the highest cognitive functions. As we move on to further discussion, these cortical areas are also related to the main neural networks, SN, DMN, and CEN, which condition social behavior. Moreover, these brain regions are phylogenetically late-developing regions of the cerebral cortex (Smaers et al., 2011). The trajectories of the neurosociological paradigm in the metaverse may be directed toward particularly pronounced functional variability in the main social neural networks, that is positively traced in long-range cortical–cortical interactions and negatively with local intranetwork variability. Functional connectivity variation has been shown to influence the anatomical variability of neural structures. Similar structural and functional variability biomarkers are found in the lateral frontal and temporoparietal regions according to the thickness of motor area layers. To a lesser extent, this is detected in the frontoparietal network (CEN) and in the DMN, in the expression of furrows (Mueller et al., 2013). For example, a well-known example of neurodiversity is left-handedness, which occurs in 10–15% of the world’s population. Interestingly, left-handedness was once debated within the social norm (Brandler and Paracchini, 2014). Such a theoretical basis for the realization of the neurosociological paradigm in the metaverse will contribute to the development of a diversity of thought and social dynamics, as well as solving such a pressing problem as the dual empathy of the neurotypical and neurodivergent people. At the same time, social neural networks interact with others main neural networks (sensorimotor network, limbic system, dorsal attention network, visual system) (Fox et al., 2006; Yeo et al., 2011; Glasser et al., 2016; Androulakis et al., 2018), and three brain subnetworks—auditory system, ventral attention network, and language network (Schirmer et al., 2012; Bernard et al., 2020; Vossel et al., 2014; Figure 2). In the concept of the social brain, the development of the neurosociological paradigm in the dynamically changing metaverse leads to the control of specific functions, and four social neural networks mediate the complex forms of social interaction between humans. The DMN, SN, CEN, and SCN mediating social cognition, motivation, and cognitive control during various interactive contexts play a key role in human social interactions.

Therefore, integrating social brain research with sociological theories contributes to a more nuanced understanding of social interactions, especially within the complex and dynamic metaverse environments.

## 3 The neurosociological paradigm

As we emphasized earlier, research on the main neural networks (DMN, SN, and CEN) of the social brain has been conducted outside the field of real social interaction since it should involve the dyads paradigm and their relationships influence behavior and/or consciousness (Feng et al., 2022). Therefore, research on intra- and intergroup social behavior is still within the paradigm of sociology as a science that studies social interaction in its reference to the present and the past (Beckert and Suckert, 2021; Abbott, 2016). The functional portrait of large-scale social neural networks (DMN, SN, and CEN) in the human brain and the understanding of social learning is widely represented in numerous studies. Still, the neural dynamics of different parts of social neural networks have not been studied in the category of social interaction (Hari et al., 2015). The main characteristics of the brain networks across human social interactions are shown in Table 1.

**Table 1:** The characters of the main neural networks across human social interactions.

The integration with the neural networks	The neurosociological side of the social neural network’s functions
Central executive network (CEN) integrates with DMN, SN, sensorimotor network, limbic system, visual system, and DAN	The dominant control neural network of the brain performing high-level cognitive tasks, integrating information from the other neural networks. CEN acts as an “external mind” in implementing the neurosociological paradigm in the metaverse, and this is particularly important in the controlled processing of information (attention), involving working memory, decision-making, organizing social behavior based on personal motives, subjective preferences and choice.
Default mode network (DMN) integrates with CEN, SN, limbic system, visual system, language subnetwork, sensorimotor network, DAN, DMN, VAN, and auditory system	DMN immediately activates after a task is completed or stopped, and there is a certain anticorrelation with CEN, which makes DMN very important in the monitoring of the internal and external environment, emotional control, and emotional memory extraction in the pauses between the neurosociological sessions in the metaverse. Increased DMN or DMN and CEN activity is associated with numerous psychiatric disorders. DMN is also referred to as the “Internal mind,” when people idly daydream or think about a new idea. The high sensitivity of DMN to cross-domain task transitions facilitates the study of the mechanisms of state transitions between the social virtual platforms.
Salience network (SN) integrates with CEN, DMN, and limbic system	SN regulates the switching between internal and external information processing involving the DMN and CEN, causing reciprocal levels of activity of these two networks in the healthy brain, which is crucial for social interaction in the dynamically changing virtual environment of the metaverse.

In the “Introduction” section of this article, we showed that the functions of the main neural networks of the social brain determined social behavior are typically investigated in the “brain-tasks” paradigm (Raichle et al., 2001; Rilling et al., 2008; Wirth et al., 2011; Tomlin et al., 2013; Elton and Gao, 2014; Gabay et al., 2014; Lisofsky et al., 2014; Fareri, 2019; Rotge et al., 2015; Wu et al., 2016; Luo et al., 2018; Yin and Weber, 2018; Delgado-Herrera et al., 2021; Feng et al., 2021; Feng et al., 2022; Zoh et al., 2022; Zheltyakova et al., 2024), which is a precursor to the development of the hyperscanning technology. This is primarily due to the neuroimaging via the fMRI, and activity of the neural network of the social brain and other main neural networks evoked in its conditions is associated with the neural system responses based on past social learning. However, dual fMRI scanning or hyperscanning technology once showed that social interaction can best be studied by simultaneously brain scanning of at least two interacting subjects inside the tomograph (Montague et al., 2002). In general, fMRI provides excellent spatial location of brain activation but limited temporal resolution (Kim et al., 1997; Schmidt et al., 2023). Therefore, the advent of hyperscanning (Montague et al., 2002) and its refinement into “brain-to-brain” technology (Hasson et al, 2012; Scholkmann et al., 2013) has made it possible to investigate the cortical interpersonal brain mechanisms underlying social interaction (Hamilton, 2021). As a result, the neurosociological paradigm of “brain-to-brain” social interaction explores brain activity in connectedness during social communication in laboratory conditions (Dikker et al., 2017; Hirsch et al., 2021; Hirsch et al., 2022; Maslova et al., 2022; TenHouten et al., 2022; Shamay-Tsoory et al., 2024). However, this opens dynamic possibilities in decoding specific socially conditioned brain algorithms and their dynamics in real-world social cognition (Nam et al., 2020; Zamm et al., 2024) and presents a modern methodology of the neurosociological paradigm in the form of hyperscanning and interbrain synchrony.

The advent of affordable mobile fNIRS-based devices creates increased opportunities to uncover the mechanisms underlying social interactions within and across generations (Moffat et al., 2024). To date, the hyperscanning method with fNIRS neuroimaging technology (Dikker et al., 2017; Liu et al., 2017; Hirsch et al., 2021; Acuña et al., 2024) and EEG (Maslova et al., 2022; Ogawa and Shimada, 2023) in free behavior has already shown its promise in the study of interbrain connectivity dynamics of the neural networks of the social brain.

Previously, we presented data on the application of fMRI to study the social brain’s maps in different testing contexts (“brain-tasks”) of the social neural networks. Despite its high spatial resolution, this method is, nevertheless, not applicable to studying social neural networks in free social interaction. fNIRS, as an artifact-free method, is used for hyperscanning in the paradigm of neurosocial interaction (Dikker et al., 2017; Balconi and Fronda, 2020; Hirsch et al., 2021; Acuña et al., 2024; Zhang et al., 2024).

The fNIRS and EEG methods have their advantages and limitations, as fNIRS and EEG have different threshold capabilities in temporal and spatial resolution scales (TenHouten et al., 2022). In combination, fNIRS and EEG allow both electrophysiological and hemodynamic brain activity data to be examined with high temporal and spatial resolution simultaneously and at the cortical location (Kaewkamnerdpong et al., 2024). The real opportunity to combine fNIRS with emerging technologies such as VR, AR, and AI not only opens a new possibility for immersive studies of brain function in clinical practice (Rahman et al., 2020) but also expands methodological possibilities in social brain research.

Neurosociological studies of the social brain in the brain-to-brain paradigm (dyads hyperscanning) are now becoming routine at the current stage of neurosociology development (Maslova et al., 2022; TenHouten et al., 2022). However, there is a dearth of brain-to-brain research based on the social brain theory and functional dynamics of the social brain. The current hyperscanning methodology in social interaction is not directly associated with the social brain theory, whose structures include the social neural networks DMN, SN, and CEN. The CEN is one of the dominant control networks in the brain, performing high-level cognitive tasks and functions alongside or in anticorrelation with the other six main neural networks (Niendam et al., 2012; Goulden et al., 2014). The CEN functions are driven by frontal (goal setting, working memory, episodic memory, awareness of complex visual information, attention, cognitive abstraction, integrative motor acts, semantics, linguistics), parietal (decision making, non-spatial attention, working memory, motor planning, speech) and temporal areas (visual awareness, visual working memory) that deactivated during speech and especially during the theory of mind (Niendam et al., 2012). Understanding the role of the CEN in reflective thought is crucial for designing the metaverse environments that promote deep social connections as a dominant control neural network performing high-level cognitive functions in integrating information from the other brain networks (Vincent et al., 2008). As a result, the CEN acts as an “external mind” in implementing the neurosociological paradigm in the metaverse, implying controlled processing of information (attention), involving working memory, decision making, and organizing social behavior based on personal motives, subjective preferences, and choice.

At the same time, the complex questions about the CEN processes (Zink et al., 2021), such as flexibility, working memory, initiation, and inhibition, previously thought to be separate processes of social behavior, may be answered in the dynamically changing metaverse based on the neurosociological paradigm.

DMN is a network of active regions during passive mental states linked to internally directed cognition including recollection of the past and thinking about the future (Buckner et al., 2008; Raichle et al., 2001). This network of the social brain has the activity pattern in undirected task states and its metabolic properties, which led to the designation of the activity observed in rest states (Raichle et al., 2001). This type of spontaneous activity relates to mind wandering and spontaneous use of recollection and future-oriented thought (Buckner et al., 2008; Mason et al., 2007). DMN immediately activates after a task is completed or stopped, and their activities have a certain anticorrelation with the CEN (Greicius et al., 2003; Lee et al., 2012). The DMN is defined as the “internal mind,” when people idly daydream or think about a new idea. The DMN high sensitivity to cross-domain task transitions facilitates the study of the neurosocial mechanisms of state transitions between the social VR platforms (Zhou et al., 2024).

Increased activity of the DMN or DMN and CEN is associated with numerous psychiatric disorders, which also opens new possibilities for the neurosociological paradigm in the modeled metaverse. Hence, understanding the DMN role in reflective thought is crucial for designing the metaverse environments that promote deep social connections and empathy among users (Luo et al., 2024).

**Figure 3:**
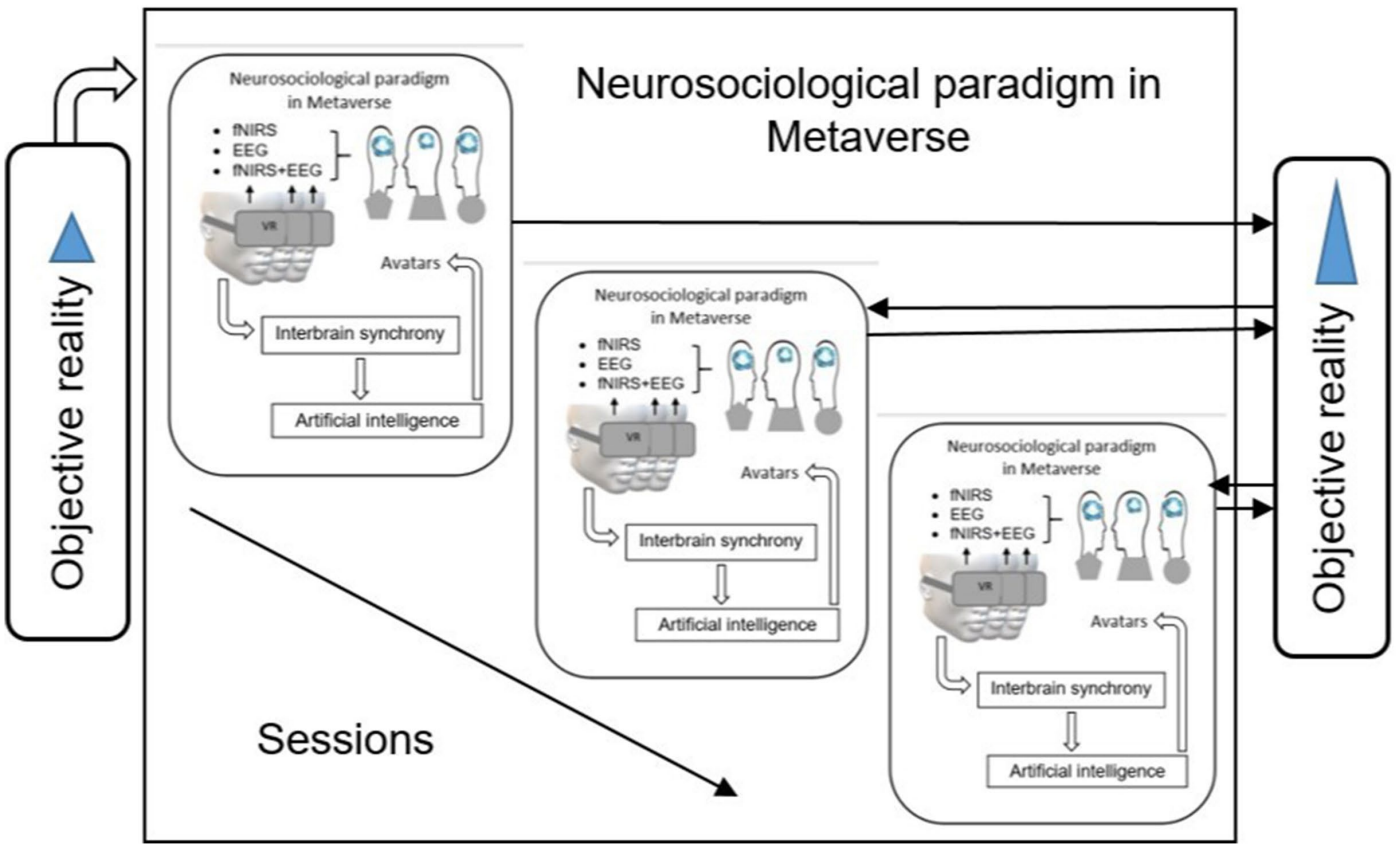
Multistage integration of the neurosociological paradigm within the metaverse, showing how EEG and fNIRS are used to monitor brain activity and achieve interbrain synchrony during social interactions. AI provides real-time feedback, guiding users through the dynamically changing virtual environments to optimize social communication. Based on our quantization hypothesis of the neurosociological paradigm, the multistage integration can represent a stream of quanta: 1. Series of quanta at the level of global interbrain synchronization of the social neural networks in the personalized metaverse for neurotypical or neurodivergent individuals. 2. Series of quanta at the level of intranetwork interbrain synchronization of the social neural networks (DMN, CEN, and SN). 3. Series of quanta of the neurosociological paradigm in the metaverse using different neurotechnologies: fNIRS, EEG, or fNIRS in combination with EEG, or using simple/complex VR scenarios.

The SN monitors the external world and carefully decides how other neural networks react to new information and stimuli. The SN moderates switching between the internal and external processing of the brain’s two main control networks: the default mode network and the central executive network (Figure 3). The SN accounts for the reciprocal activity levels of these two neural networks in the healthy brain, which is critical to understanding the SN role in designing the metaverse environments that promote deep social connections (Elton and Gao, 2014; Goulden et al., 2014).

## 4 The social neural networks’ basis of the neurosociological paradigm

By now, the main compartments of the human social brain have been well studied in the context of different types of social interaction, in which the neuronal nucleus of the DMN, SN, CEN modulus, and the subcortical network (SCN) of the reward are activated or inhibited (Feng et al., 2021). In the context of social interaction that induces social pain—due to social threat, exclusion, rejection, loss, or negative evaluation—it activates the neural networks of the ACC (Rotge et al., 2015). At the same time, different types of social tasks activate different subregions of ACC neural networks. In general, it has been found that the set of cognitive functions mediated by ACC includes cognitive concentration and working memory when performing tasks or tests, behavioral decision-making, analysis of internal and external states, emotional and motivational displays, and evaluation of the meaning of social information. ACC is a subnetwork of the DMN, which mediates human social cognition (Feng et al., 2021).

At the same time, the DMN plays a coordinating role with the other main neural networks of the brain during the passive sensory processing (Greicius et al., 2003; Andrews-Hanna et al., 2014). For example, the coordination of the DMN with the brain’s visual network is enhanced when the mind subconsciously evaluates an aesthetic beauty (Vessel et al., 2019), especially when perceiving outstanding works of art or architecture. The DMN, along with the language subnetwork, is involved in semantic processing when encoding or translating meaning into spoken or written words. Semantic brain regions (dorsal medial prefrontal cortex, anterior cingulate cortex, retrosplenial cortex, angular gyrus, middle temporal gyrus, anterior temporal region, and right cerebellum) demonstrate spatial and functional involvement in the DMN (Wirth et al., 2011). The DMN interacts with the limbic system to process or evaluate the personal emotions and emotions of others (Raichle et al., 2001). The DMN is involved in modulating other people’s feelings, intentions, and traits to model, explain, and predict the behavior of others (Krueger et al., 2009; Menon, 2023). Social comparison is ubiquitous in social interaction and significantly impacts on people’s well-being and decision-making. According to neuroimaging data, this type of social interaction according to neuroimaging data (Luo et al., 2018) is driven by activation within several neural networks in the brain, which are nevertheless related to the main socially conditioned neural network, namely, the SN (Elton and Gao, 2014). The functional complexity of the DMN social role, responsible for the above cognitive processes, necessitates a multidimensional application of the neurosociological paradigm in the metaverse to enhance and/or develop social interaction.

The main functional fields of the SN are located within the anterior cingulate, the anterior insula (Seeley, 2019; Uddin, 2014), and in the presupplementary motor areas (Bonnelle et al., 2012). The SN also includes neural structures in the amygdala, hypothalamus, ventral striatum, thalamus and brainstem nuclei, ACC, medial temporal network, parahippocampal gyrus, olfactory lobe, and ventral tegmental areas (Luo et al., 2018). Moreover, the ventral striatum and ventromedial prefrontal cortex are activated depending on the direction of social comparison from the SN subnetworks in the process of downward social comparison, and in upward social comparison, consistent involvement of the anterior insula and dorsal anterior cingulate cortex.

The most important decisions are made in the context of social interactions because people live and interact in very complex social environments (Rilling et al., 2008). A model example of social interaction is the Ultimatum Game (UG) (Gabay et al., 2014). The UG is a task often used to study social decision-making and originates from behavioral economics (Gabay et al., 2014). In this task, different compartments of the socially conditioned SN are activated when seeking a fair solution. In response to unfair offers compared to fair offers, consistent activations were seen in the anterior insula, anterior mid-cingulate cortex, ACC, and medial prefrontal cortex. In contrast, in response to injustice during the UG participation, there is sequential activation of the bilateral mid-anterior insula, anterior mid-cingulate cortex/ACC, medial supplementary motor area, and cerebellum (Gabay et al., 2014). In social interaction, trust and reciprocity are relevant to all human interactions and facilitate cooperation (Vilares et al., 2011; Bellucci et al., 2016). Trust is associated to some degree with a healthier, more egalitarian, and productive society (Krueger et al., 2007).

This context of social interaction activates many of the neural networks underlying trust, reciprocity, and feedback learning (Gabay et al., 2014). The response activation occurs in the anterior insula during trust decisions in the one-shot of the investment game (IG) and decisions to reciprocate in the multiround IG, likely related to representations of aversive feelings. However, at the feedback level, other neural networks, such as the intraparietal sulcus, are engaged in the context of trust learning, and in the multiround the IG permanently activated the ventral striatum, associated with erroneous expectation of reward. Model-based analyses show that the choice to trust is based on a differential evaluation of reciprocity depending on social proximity to the partner. This critical social evaluation is encoded in the neural networks of the ventral striatum and medial prefrontal cortex (Fareri, 2019), which are subnetworks of one of the key social neural networks, namely, the SN (Seeley, 2019).

In the context of social conformity, human social behavior often aims to agree with the group opinion (Wu et al., 2016). This form of social interaction is related to the functions of the ventral striatum, dorsal posterior medial frontal cortex, and the anterior insula, which are part of the SN—social neural network. It is assumed that among the main brain and the subnetworks, there is a “common” neural system consisting of the dorsal posterior medial frontal cortex and the anterior insula to detect deviations from the group norms to facilitate the adjustment of behavior by the normative opinions (Montague and Lohrenz, 2007; Tomlin et al., 2013; Xiang et al., 2013). The functional interaction between SN and CEN is that an increase in SN information activity initiates the generation of a control signal in CEN, which switches this neural network to monitor attention and perform executive control (Menon and Uddin, 2010).

Therefore, the salience network, responsible for detecting and filtering stimuli, can be a key in adapting the virtual environments to enhance user engagement and focus, particularly in scenarios requiring rapid decision-making.

Social interaction in the context of social cooperation based on kinship and reciprocity is characteristic of primates and humans. Cooperation is an essential component of human social behavior. As such, humans are unique in their ability to represent shared goals and self-regulate to comply with and enforce cooperative norms on a large scale (Zoh et al., 2022). Brain neuroimaging under enforced cooperation has shown that the social neural networks primarily conditioning reward responses, namely brain regions such as the striatum and the orbitofrontal cortex, are involved in processing (Bhanji and Delgado, 2014). In contrast, disruption of different types of cooperation (with self and others), caused by the manifestation of negative emotions, activates neural networks of the insular cortex (Yang et al., 2019), representing a subnetwork of the DMN. In human social interaction, deception is a ubiquitous behavior that plays a vital role in everyday life and is found in a wide range of situations (Lisofsky et al., 2014). The neurobiology of deceptive behavior is well understood and is represented by the involvement of the primary neural networks (Zheltyakova et al., 2024). This has been shown in numerous neuroimaging studies about error detection, attention shifting, image recognition, inhibition, components of decision-making, language, which is accompanied by activation of the social brain’s regions (Vendemia and Nye, 2018). Thus, the angular, inferior frontal, and postcentral gyrus are activated in differentiation lying from truth-telling (Feng et al., 2022). This network includes the bilateral prefrontal cortex, left middle frontal gyrus, insular cortex, right ACC, inferior parietal lobe, and intraparietal sulcus (Christ et al., 2009).

Moreover, prefrontal cortex activation is positively correlated with lying frequency across individuals (Yin and Weber, 2018), and lying is associated with activity in the left caudate, ventromedial prefrontal cortex, right inferior frontal gyrus, left dorsolateral prefrontal cortex (Yin and Weber, 2018). In summary, the extensive network of brain regions involved in the deception process includes the prefrontal cortex, insular cortex, ACC, and inferior parietal lobule. It is related to the two main social neural networks—SN and CEN—which mediate multiple social forms (Feng et al., 2022). Moreover, during lie preparation compared to lie execution, the specific areas in the superior parietal lobe become more active (Zheltyakova et al., 2024). Consequently, deception as a form of social interaction is due to the involvement of many cognitive systems (attention, memory, motivation, emotion) in the activity, but these neural systems are not exclusive to deception and are not universally involved in all forms of deceptive behavior (Vendemia and Nye, 2018; Feng et al., 2022). The social act of deception has been associated with activation in various neural networks mediating more than just social behavior (Lockwood et al., 2020). Moreover, the ACC, as an SN subnetwork, is important and involved in performing deception in tasks with high ecological validity (Delgado-Herrera et al., 2021; Zheltyakova et al., 2024).

Neuroimaging studies have revealed a vast network of brain regions involved in the deception process, including the prefrontal cortex and inferior parietal lobule as the CEN compartments, and the insular cortex and ACC as the SN compartments. In this context, we should mention the unique CEN function in social interaction as a key top-level neural network for controlling social behavior. Based on incoming data from other networks, the CEN processes various information such as social flexibility, working memory, initiation, and inhibition, which were previously considered separate processes but now are the neural network controlling social behavior (Niendam et al., 2012).

Thus, different types of social interactions are carried out under the control of the shared neural networks, DMN, SN, and CEN, which mediate social cognition, motivation, and cognitive control in the different interactive contexts. Large-scale neural networks are also involved in the hierarchical information processing related to social interactions (Alcalá-López et al., 2018; Schurz et al., 2021). For example, the DMN is involved in modeling other people’s feelings, intentions, and traits to explain and predict other people’s behavior (Krueger et al., 2009). The SN and SCN modules contained bilateral striatum are relevant to the general motivational system that encodes the reward/punishment properties of social options and outcomes, considering not only self-interest but also normative social principles (Luo et al., 2018; Montague and Lohrenz, 2007). The CEN is involved in integrating information encoded in the DMN (mental states) and SN (motivational salience) to optimize social behavior (Buckholtz and Marois, 2012; Krueger and Hoffman, 2016; Krueger et al., 2020). The CEN, the brain’s external mind and the dominant network controlling task selection and behavior, uses data from other neural networks.

Since knowledge about the interactions between the brain systems that transiently change according to the patterns of social interaction is crucial for studying the plasticity of standard cognitive control (Cocchi et al., 2013), then the extraordinary possibilities of the neurosociological paradigm in the metaverse offer innovative scientific and applied perspectives in this direction. The critical brain networks across human social interactions are shown in Figure 2.

The interneural network interaction results in an assessment of internal drives and personal preferences, which ultimately guides the individual’s choices. Furthermore, in neuroimaging studies, the underlying social neural networks represent the neural correlates of consciousness as emphasized by Crick and Koch (1990), making neurosociology a basis in studying conscious social learning and developing the neurobiological theory of consciousness.

To date, the majority of the studies of the primary neural networks (DMN, SN, and CEN) of the social brain have been conducted outside the field of real social interaction. The neurosociological paradigm in the metaverse is extremely promising in decoding specific social algorithms of the brain and their dynamics in socially connected behavior. It can reconsider the most relevant issues of sociology as a discipline from the innovative perspectives in the present and the future. Thus, the neurosociological paradigm in the metaverse based on the concept of the social brain and main social neural networks presents unprecedented opportunities for both neurotypical and neurodivergent individuals in their future social interactions.

## 5 Forward to the metaverse

It is widely recognized that the metaverse represents an opportunity to extend the physical world by applying technologies to seamlessly interact with real and simulated digital environments (Dwivedi et al., 2022). Following this logic, it can be argued that the metaverse represents an opportunity to infinitely extend neurosociology’s ability to study unimpeded social interaction in the transition from reality to the immersive environments and in the virtual movement between the metaverse digital spaces. The absence of borders in the metaverse carries crucial social, economic, and geopolitical implications (To et al., 2024). In doing so, neurosociological research within the new concept of hyperscanning represents an environment that will enable the metaverse to be developed as a sustainable virtual society, even though the metaverse can motivate users and create a new digital society with feedback on the effects on social interaction in the real world (Limano, 2023).

AI can provide a limitless option for social accessibility of the metaverse by creating the realistic avatars, new digital products and services, and facilitating remote work and collaboration in different areas, including data science (Soliman et al., 2024). The characters in the virtual world should be in constant development with the users and react dynamically to unexpected social interaction situations, which can be a function of AI (Thakur et al., 2023).

In the metaverse, the neurosociological idea unites social interaction of people, avatars, and holographic images in the virtual world. The responses of social interaction in the metaverse will be reflected in the functional dynamics of the social neural networks and the specifics of regulation of the executive functions. Based on the above, we believe that all forms of social interaction in the metaverse will be a product of the social brain. This is the fundamental basis and a new goal for neurosociological studies of social interactions in the metaverse multicompartment digital space. However, it should be realized that while the metaverse provides ample opportunities for social interaction, ethical concerns such as data privacy, the digital divide, and the potential for social isolation must be carefully considered and addressed.

The metaverse can be characterized by four compartments, each representing a level of realization of the neurosociological paradigm. The first compartment is the environments which include realistic, unrealistic, and fused virtual environments. The second is the interface point of view, such as 3D, immersive, and physical methods. The third compartment is interaction as social networking and collaboration. The last one is persona dialogue in the metaverse social platform. These metaverse compartments have many degrees of freedom and, therefore, go beyond the realistic environments. It is important to highlight that social value is a key consideration when evaluating the worth of the metaverse related to the new benefits to society as a whole. The point is that the metaverse focuses on the interactions that, firstly, go beyond the conversations between users and non-playable characters. Second, the metaverse involves redefining the social meaning of the metaverse as a 3D society rather than a copy of the real world (Dwivedi et al., 2022). As a result, the virtual world is more than an attractive alternative sphere for human socio-cultural interaction (Dionisio et al., 2013). No matter how complex the authors present the development of the metaverse (Dwivedi et al., 2022; AbuKhousa et al., 2023), including in terms of information interplanetary platforms (Vanderdonckt et al., 2024), we believe that this will be the main social development of humanity. Although in its infancy, the development and even survival of the metaverse are human centric (Mourtzis et al., 2022).

The theoretical, methodological and experimental justifications of the neurosociological paradigm can accelerate the development of the metaverse. Whereas previously the virtual worlds were dominated by the platforms, such as Second Life, Cryworld, Utherverse, IMVU, and World of Warcraft, the metaverse as a new Internet technology is processing its development through a collaboration of giant companies, such as Epic Games, Meta, Niantic, Nvidia, Microsoft, Decentraland, and Apple (Buana, 2023).

Many authors believe that the key factors in the metaverse development are the new capabilities of devices, such as headphones/headsets and AR and VR glasses (Xie et al., 2021). The metaverse utilizes not only these devices but also blockchain technology and avatars as a part of a new integration of the physical and virtual worlds (Lee et al., 2021; Liu and Steed, 2021). The AR, VR, amd XR headsets and glasses create an effect of presence or immersion, represented in the metaverse to a greater extent than the operation of VR devices in the 3D environment (Dwivedi et al., 2022).

The VR headsets are opaque and block the surrounding space when used. In contrast, the AR-based devices are transparent and allow users to see the surrounding space in front of a person with an additional image projected onto it. After combining the possibilities of digital spaces generated by AR/VR into a new spectrum of reality-altering technologies, MR has emerged. In the MR experiences, the user can interact with both digital and physical elements in different conditions of the experience (Martens et al., 2019; Bouzbib et al., 2023; Salatino and Burin, 2024). The term “extended reality” (XR) defines VR, AR, MR, and any technology that blends the physical and digital worlds (Chhabhaiya et al., 2024). This virtual continuum is currently the basis of the extended metaverse (Alpala et al., 2022; Shin et al., 2024). In terms of technical and programmatic capabilities, VR, AR, and MR have already enabled the transition from two-dimensional (2D) to 3D. Still, they do not replace the existing digital platforms but enhance them by merging with the current Internet infrastructure to encourage a more diverse form of interaction, predominantly through today’s smart devices (Cho et al., 2023).

Literature reviews show that transitioning from 2D to 3D (VR technology) is a useful tool in different applications and fields (Hamad and Jia, 2022). Notably a large panorama of the effective VR application has been demonstrated in the numerous studies of social cognitive training in the autistic neurodivergent group (Kandalaft et al., 2013; Didehbani et al., 2016; Liu L. et al., 2024). The literature analysis of the VR application shows that the traditional practice of improving VR-based social communication and social cognition skills in neurodivergent subjects presents a multicomponent model (Didehbani et al., 2016). Essential blocks of the model are the pre- and postmeasures, VR environments, demographic variables of the participants, intervention design, and the avatars represent a user in the virtual world, which were modeled to resemble each participant. This model has been successfully replicated to achieve the practical goals (Kandalaft et al., 2013; Didehbani et al., 2016). Combining new advances in VR, AR and MR with AI is a more innovative level of the immersive Internet-based digital landscape and massively multiplayer online games. Such integration of technologies ensures the development of the social and physical activity of subjects in VR, considering the multifactor nature of social motives and the large array of data obtained. These technologies use VR platforms and AI to extract categories or clusters of responses automatically. The effectiveness of the VR and AI integration (Sarupuri et al., 2024) indicates the key role of VR platform enhancement, big data analytics, and AI in the metaverse development. This is equally true for the role of blockchain technology (Elsadig et al., 2024; Huawei et al., 2023; Lee et al., 2023; Soni et al., 2023; Bashir et al., 2023), a discussion of which is beyond the scope of this study.

Thus, we can generalize that the neurosociological paradigm in the metaverse, whose different components now have been successfully proven in experimental and applied neuroscience, according to the references cited, represents the integrated technological system. As shown in our study, the theoretical foundations of the neurosociological paradigm in the metaverse are the symbolic interactionism theory and Piaget’s theory of genetic epistemology. The methodological basis of the neurosociological paradigm in the metaverse is represented by technologies of interbrain hyperscanning (dyads and more individuals) and phenomena of synchrony of electrical and/or metabolic activity of the main social neural networks, as well as technologies of registration of the executive functions’ synchrony. The digital technologies of the system are represented by the socially personalized VR platforms and automated big data analytics technologies using real-time AI and feedback based on the AI control of the metaverse.

### 5.1 The neurosociological paradigm in the metaverse for development of social communication

**Figure 4:**
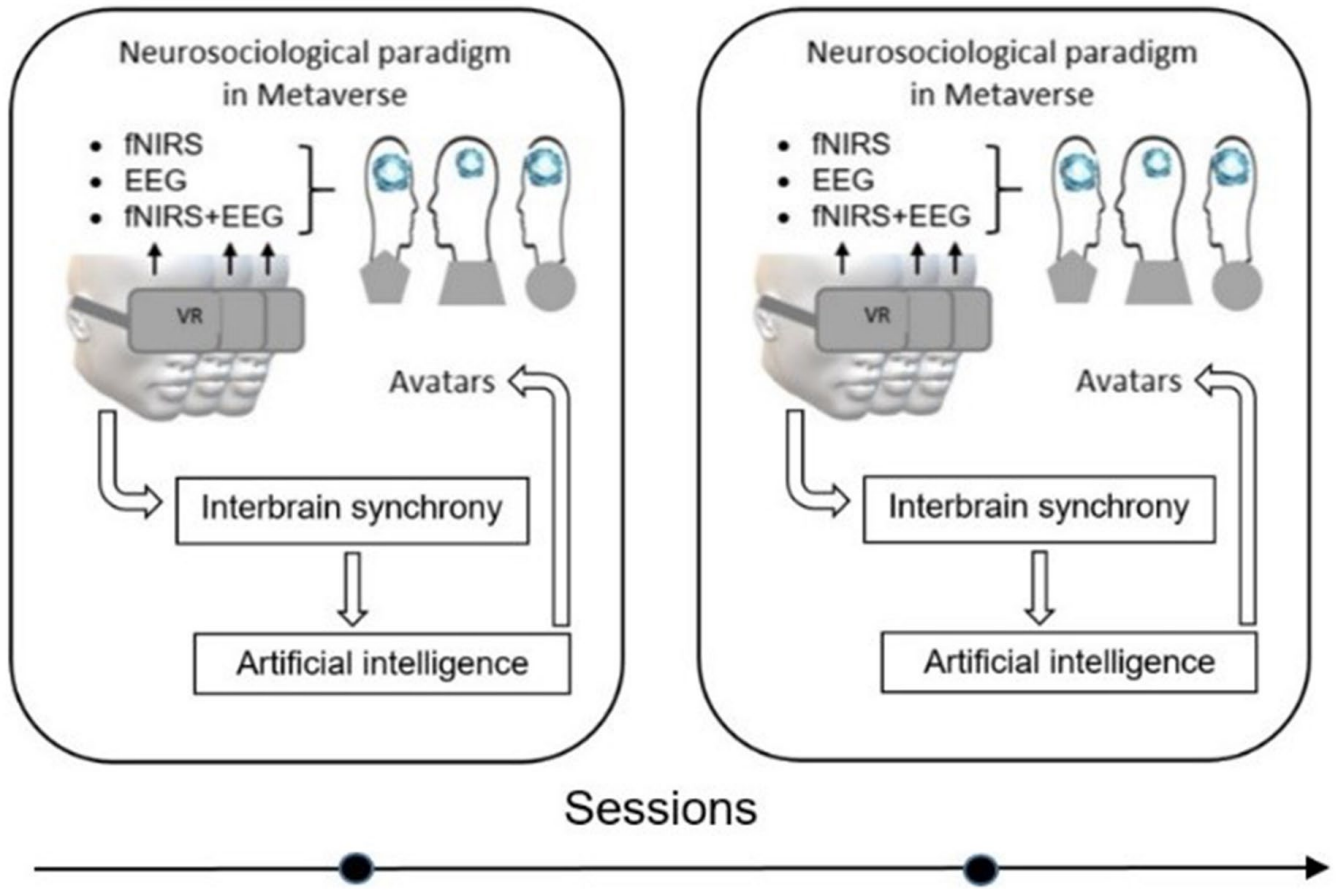
Implementation of the neurosociological paradigm in the metaverse, demonstrating how users engage with the VR platforms through EEG/fNIRS neurotechnologies. AI dynamically adjusts interactions based on real-time interbrain synchrony, creating a continuous feedback loop between virtual experiences and real-world cognitive processes.

The metaverse digital space within the symbolic interactionism theory framework is considered a virtual environment of social interaction and human activity. Based on Piaget’s theory of genetic epistemology we can predict that interaction with the metaverse virtual world will form a new cognitive structure of social neural networks, the biomarker of the formation of which will be interbrain synchrony of electrical (EEG-based) and/or metabolic (fNIRS-based) activity of the main social neural networks (CEN, DMN, and SN). On the neurophysiological basis of interbrain synchrony because of socially motivated activity in the metaverse, a new homeostasis of social interaction will be formed, and both empathy and dual empathy will be objectively solved, which cumulatively will improve the personal experience of social interaction in the post-metaverse behavior (Figure 4).

Section 1.5 in this article discusses about developing the neurosociological paradigm in the metaverse can be viewed as a three-phase process. Realization of one of these processes, namely, the real-time monitoring and feedback, is demonstrated in Figure 4 in the form of two frames when interbrain synchrony as the quant of the successful social interactions serves as a critical feedback mechanism between the social neural networks and the metaverse environments by AI. Therefore, we propose the hypothesis of quantization of the neurosociological paradigm in the metaverse as a theoretical framework to explain the realization of a social goal within a single virtual frame/session in the metaverse. The quantization of the neurosociological paradigm in the metaverse can be a result of social interaction both within one virtual social platform and as a result of changing virtual domains, which allows personalizing the structuring of the metaverse for use in the neurodivergent groups of people.

Figure 3 shows the quantization flow of the neurosociological paradigm in the metaverse. As a result, the integrated metaverse digital space through AI-enabled real-time feedback on the parameters of the users’ cognitive, neurophysiological and emotional states will be the basis of their personalized social experience (Guan and Morris, 2022). Meanwhile, the inclusive environment created in the metaverse should be informationally richer and more personalized than the objective reality of the environment (Yin et al., 2022).

The neurosociological paradigm in the metaverse based on AI control of the social VR platforms and neurophysiological processes of interbrain synchrony presented above will allow the neurodivergent groups, which are characterized by different ways of brain function, information interpretation, and environmental perception (Dwyera, 2022), to use the personalized profiles of the VR platforms during hyperscanning to achieve the controlled interbrain synchrony and get personal socially beneficial adaptive behavioral outcomes.

The potential for neurodivergent applications is promising. For neurodivergent people, the progressiveness of immersive environments is due to their high interactivity, which is positively reflected in the development of imagination and role learning, which is typical in real social situations (Lorenzo et al., 2016).

The general view of VR about the education of children with disabilities correlates with its inclusiveness, informativeness, and accessibility to information environments that are virtually inaccessible to some of them (Chițu et al., 2023). The greater accessibility of the learning environment in VR is about the development of social and emotional abilities (Didehbani et al., 2016; Fromm et al., 2021).

This is achieved by the fact that the metaverse opens up for neurodivergent groups of children and adults with huge possibilities of fast social communication and imitating the inner world through advanced 3D images and avatars. It is important to recognize that the neurodivergent states appear to be natural differences in the functioning of the human brain, not an aberration that, therefore, does not need to be “fixed” or “corrected” (Milton, 2012). The metaverse’s passional advantage over the real world is its multiplatform capabilities (Alhasan et al., 2023).

The metaverse, as a set of social platforms with a large community and many virtual opportunities to build a personalized education strategy and express their creativity and scenarios, is available to other virtual community participants. At the same time, the metaverse is always connected with real life. The modern metaverse strategies for the neurodivergent groups provide social interaction through gamification and collaborative learning (Han et al., 2023; Lampropoulos and Kinshuk, 2024), and text and voice communication, which is a special feature for the development of cognitive and motor skills, further facilitating social interaction (Du et al., 2021). Finally, the metaverse, due to its unique properties of interpersonality into opening up opportunities for building social interaction, can successfully address the well-known dual empathy issue (Crompton et al., 2021; Milton, 2012; Milton et al., 2022).

The integration of the pioneering neurosociological paradigm in the context of the metaverse emphasizes its potential to revolutionize our understanding of social interactions by leveraging advanced methodologies such as hyperscanning, interbrain synchrony, and interpersonal empathy. The neurosociological paradigm with hyperscanning and interbrain synchrony technology in the metaverse provides the objective biomarkers for achieving successful social interactions to the interacting users (two or more subjects) at each stage of quantizing their behavior. In this sense, the integrative function of the neurosociological paradigm can be defined as a moderator of the provided capabilities of the metaverse platforms at the points of transition of the cognitive states during the moments of interbrain synchrony. The moderating role of the neurosociological paradigm can only be realized in integration with AI, which is the metaverse booster for users in achieving their social and behavioral outcomes.

AI increasingly intertwines with VR, creating a more personalized and intuitive user experience. The advantage of AI-based VR is the ability to analyze user behavior and preferences, subsequently adapting the virtual environment to individual needs. The integration of AI will lead to significant progress in the future of VR. We believe hyperscanning technology combined with AI in the metaverse will represent the key feedback between users and digital spaces. It all comes down to the fact that analytical predictions link the relevance of AI for the metaverse to the importance of big data processing to improve immersive experiences in virtual digital environments and provide human-like intelligence to the virtual agents (Huynh-The et al., 2023; Alotaibi, 2024; Zawish et al., 2024). As a result, the metaverse appears as a hypothetical upcoming Internet iteration that will support decentralized, long-term, three-dimensional virtualized online environments between the financial, virtual, and physical worlds that are becoming increasingly connected (Hovan George et al., 2021).

In the context of this paper, we focus on an essential aspect of XR, such as the ability of people to interact and communicate with each other in real time using VR technology. Combining XR with social interaction creates a VR perspective for people worldwide to communicate. The emergence of social VR platforms has become one of the fastest growing trends in VR. People can interact with each other in real time in a virtual environment, host parties, attend concerts, and play multiplayer games. Social VR platforms are becoming increasingly user-friendly, diverse, and community-oriented, evolving toward a more inclusive social future of VR (Dzardanova et al., 2024). In this aspect, the neurosociological transformation of the social VR platforms based on the neurosociological paradigm in the metaverse offers a great opportunity for studying the world’s social flows and improving the metaverse. At the same time, a special goal of the social VR development in shared virtual spaces will be human-to-human communication and interaction, avatar creation, and personalization, which require an AI component of the metaverse (Huynh-The et al., 2023; Alotaibi, 2024; Zawish et al., 2024).

### 5.2 Empirical vision of the neurosociological paradigm in the metaverse

It should be noted that, at present, the merging of the physical and virtual worlds is not yet achieved without a gap between the digital and physical realities (Shen et al., 2022). Still, this merger will eventually turn the metaverse into an ideal social experience, regardless of the neurodivergence of social groups and the different motivations of users.

At this advanced stage, the social metaverse will be the mixed digital space of integrated realities. The integration goes beyond the traditional human visual experience as it will include the heteromodal sensations (Pham et al., 2022; Jot et al., 2021), the Internet of Things (Guan and Morris, 2022), digital twins (Tu et al., 2023), wireless non-invasive and invasive neurotechnological devices (Bozgeyikli, 2021; Alsamhi et al., 2023; Bashir et al., 2023), and biometric sensor technologies (Wang et al., 2023). This exploration promises to redefine human lifestyles and work paradigms. From a functional perspective, interoperability in the metaverse will be a universal digital ecosystem with social interaction in the form of seamless and secure information sharing and interactions between different systems or platforms, supported by consensus and common standards (Dionisio et al., 2013; Hyun, 2023; Behr et al., 2023).

#### 5.2.1 Hyperscanning perspective

The hyperscanning interbrain synchronization has identified the regions of the association cortex (frontal, parietal, and temporal lobes) that form the crucial CEN whose function accounts for the interbrain coupling, according to available data from the fNIRS and/or EEG (Dikker et al., 2017; Liu et al., 2017; Wang et al., 2018; Barde et al., 2020; Feng et al., 2021; Hirsch et al., 2021; Schwartz et al., 2024). Furthermore, it can be argued that hyperscanning and interbrain synchrony is a new aspect in the development of modern sociology, namely neurosociology, which investigates the neurophysiological mechanisms of the social brain functioning in the dyads and more individuals.

#### 5.2.2 Face-to-face and interbrain coupling perspective

The fact is the literature emphasizes the crucial role of face-to-face interactions to enhance the superior mode of communication for interpersonal connection (Hirsch et al., 2021; Schwartz et al., 2024). Mechanisms of interbrain coupling in the face-to-face interaction paradigm have received empirical evidence in many studies as one of the highly effective technologies for multifaceted solutions to the challenges similar to human challenges, main technological obstacles, and process challenges arising when using the metaverse (dos Santos et al., 2024). The literature on the empirical research in the subject area of our study actualizes the methodological revolutionarity of the neurosociological paradigm in the metaverse based on hyperscanning as a neurophysiological process of interbrain communication, which will be crucial for future metaverse initiatives. In support, we describe the empirical evidence below.

The empirical evidence for the link between traditional face-to-face interactions and interactions in the metaverse comes from musician hyperscanning data showing a high level of synchrony of dual intersound paired pianists’ brain activity, which is hypothesized to result from the integration of external and internal sources of auditory information. At the same time, the neurophysiological mechanisms of interbrain synchrony in pianists were in alpha oscillations by the EEG data (Novembre et al., 2016).

The key role of the increased alpha-band in the interbrain synchrony as a predictor of the interpersonal behavioral synchrony across the participants is also shown in the study of the stimulatory effect of oxytocin on enhancing interbrain synchrony during social coordination in male adults (Mu et al., 2016). Alongside this, it is known that synchronization in the alpha and beta bands does not appear to be mediated by physical properties of speech but arises directly from the dyads (face-to-face) interactions (Pérez et al., 2017). These results emphasize that the neural basis of linguistic communication and the social essence of verbal communication should represent one of the bases for implementing the neurosociological paradigm in the metaverse.

A multipersonal learning situation with high levels of social dynamics can also be transferred to the metaverse on the basic condition that EEG portable headsets in a school classroom support interbrain synchrony data when many stimulus properties of all parties (teacher/students) are combined. For example, experience (Dikker et al., 2017) shows that face-to-face interaction before the class starts is a kind of “activator” of brain-to-brain synchrony between students during the study session. Consequently, in scenarios of the neurosociological paradigm realization in VR space, social dynamics in the metaverse within the face-to-face paradigms represents host real-time multisensory social interactions between two or more people that occur synchronously and involve the multiple senses sensor technologies and feedback of the neurosociological paradigm.

Hypothesizing an empirical link between traditional face-to-face interactions and interactions in the Education metaverse, it should be emphasized that brain-to-brain synchrony and learning outcomes vary by student-teacher dynamics (Bevilacqua et al., 2024). Thus, interbrain synchrony in students is significantly higher during a video than a lecture, and student engagement and teacher likeability during a lecture. Consequently, the social factors reflected on brain-to-brain synchrony in the real-world settings of the study group can be used in the neurosociological paradigm of the metaverse to predict, for example, cognitive outcomes of academic performance.

Interbrain synchronization without face-to-face communication also occurs during co-operative social engagement, which increases with improved team performance (Reinero et al., 2021). The study shows the potential role of interbrain synchrony in collective performance and intergroup interaction, which will be widely presented in the metaverse at the level of problem-solving tasks, VR games, and the team members’ brain dynamics. We believe that the neurosociological paradigm can be successfully implemented in the metaverse based on interbrain synchrony, which according to Reinero et al. (2021), can serve as an implicit measure of predicting the team success or assessing the connectivity between neurodivergent subjects and/or groups.

The Musical metaverse as a multiuser musical environment holds significant potential for music-making in music composition and performance through virtual environments based on technologically mediated social interaction. Hyperscanning in conditions of musical improvisation, shows a higher number of main brain networks active at the delta (2–3 Hz) and theta (5–7 Hz) frequencies in the interbrain connectivity, and the difference of the activated networks, primarily CEN and SN between the two guitarists. This points to the brain regions that implement mechanisms and allow individuals to engage in the temporal coordination of cooperative actions (Sänger et al., 2012; Muller et al., 2013). Consequently, neurosociological hyperscanning technologies in the “Musical Metaverse” have great potential in investigating the neurophysiological basis of complex interpersonal coordination of actions in musical art. Interbrain synchrony in the delta and theta bands is mediated by speech signals in traditional hyperscanning (Pérez et al., 2017) and can be a biomarker of successful interbrain coupling in the metaverse.

An important aspect of the empirical experience from hyperscanning data in the metaverse is the high neural synchrony in romantic couples compared to unfamiliar pairs of people (Kinreich et al., 2017). Under these conditions, interbrain synchrony occurs in the temporoparietal regions of the social brain. It is manifested by a gamma rhythm that is conjugated with nonverbal social behavior and amplified during the moments of eye-to-eye interaction.

The empirical studies of a higher behavioral form, such as speech, show more interbrain synchrony in the “human–human” than “human–machine” tasks. Moreover, the same main neural networks of temporal and lateral-parietal regions are synchronized in the range of theta/alpha (6–12 Hz) EEG rhythms (Kawasaki et al., 2013). Interbrain synchrony in the delta and theta bands is also mediated by a speech signal in hyperscanning (Pérez et al., 2017). It can be a biomarker of the success of interbrain coupling in the metaverse. These results suggest that interbrain synchronization is closely related to speech synchronization between subjects and can be a major strategy of the neurosociological paradigm in the metaverse.

Finally, interbrain synchrony of brain electrical activity across multiple frequency bands can occur without physical presence or direct visual or auditory information about the participant in the interaction (Wikström et al., 2022). At the same time, the current capabilities of hyperscanning go far beyond the dyads paradigm (Maslova et al., 2022). Overall, this indicates the potential for interbrain synchrony to positively influence social relationships in a distributed network of the different metaverse platforms.

Spatial localization of neural networks using fNIRS hyperscanning technology showed the patterns of synchrony in the metabolic activity of the social brain networks in frontal, temporal, and parietal cortical regions in the face-to-face “parent–child” relationships as well as in romantic couples (Zhao et al., 2024). It is important to emphasize that the increased neural interactions in the brain regions mentioned above highlight their involvement in cognitive functions during the prolonged interaction with close partners and affection, benefiting from cooperation and collaboration.

Thus, the hyperscanning of traditional face-to-face or interbrain coupling and the real technological possibilities of transferring such coupling to the metaverse by fNIRS and EEG actualizes the overall methodological revolutionary nature of the neurosociological paradigm for the metaverse and makes it crucial for future metaverse initiatives.

In the metaverse, the neurosociological idea unites the social interaction of people, avatars, and holographic images in the virtual world. The responses of social interaction in the metaverse will be reflected in the functional dynamics of the neural networks of the social brain and the specifics of regulation of the executive functions. Based on the above, we believe that all forms of social interaction in the metaverse will be a product of the social brain, a fundamental basis and a new goal for neurosociological studies of social interactions in the metaverse digital space. From this point of view, hyperscanning by the fNIRS-based neuroimaging method or wired EEG-based is now able to reveal the phenomenon of interpersonal neural synchrony in the situations of social interaction (Zhao et al., 2024). According to the hyperscanning data, synchrony of neural networks plays a central role in establishing social connection or performing collective social action in social behavior (TenHouten et al., 2022). Typically, synchrony occurs within the prefrontal cortex in different contexts of social interaction, and/or synchrony captures subregions of the frontal cortex. Thus, synchrony during social behavior is primarily recorded in the brain’s large-scale neural network CEN which controls the main social neural networks. The fNIRS-based hyperscanning in neurosociology also relies on synchrony (Czeszumski et al., 2020). Thus, the involvement of the social neural networks (DMN, SN, CEN) in social interaction remains a poorly explored area. Still, they are available for hyperscanning in the neurosociology paradigm and even more so in the metaverse. The EEG method of recording the electrical activity of cortical fields and its combination with fNIRS (Wang et al., 2024) is currently the most informative approach in studying the main social neural networks, as well as the main controlling neural network of the brain—CEN, but also from the perspective of interpersonal social synchrony. Recently, interbrain synchronization has been shown in the mixed-reality environment (Ogawa and Shimada, 2023), which is known to be the environment through which the metaverse can be entered. The transformation of the neurosociological paradigm from the real world to the virtual metaverse is showed in Table 2.

**Table 2:** Transformation of the neurosociological paradigm from the real world to the virtual metaverse.

The fields of social sciences	Methods and technologies	Science objects	Social communication
Sociology	Participant observation; non-participant observation; longitudinal study; surveys; interview; questionnaires; focus-group; sociological VR	Society, culture, and people; large populations with a manageable investment of time, effort, and money; a conversation between a researcher and respondent	Postsocial communicative experiences
Neurosociology	Hyperscanning, fMRI	Hyperscanning; social brain imaging; dyads or mini group	Model of social communication” brain-tasks”
	Hyperscanning, fNIRS, EEG	Hyperscanning; dyads or groups	Interbrain social imaging; interbrain social imaging synchrony and EEG synchrony; real social interpersonal communication
Neurosociology in VR	VR, Hyperscanning, fNIRS, EEG, BMI, body multimodal technologies, AI	Hyperscanning; dyads or groups	Interbrain virtual communication; interbrain synchrony; interfunctional synchrony; dyads or groups in VR social communication
Neurosociology in the metaverse	VR, AR, XR, hyperscanning, fNIRS, EEG, BMI, body multimodal technologies, AI	Hyperscanning; social groups, intercontinental groups, interplanetary groups, AI feedback metaverse control, BMI enhancing fNIRS, EEG, body multimodal technologies	Interbrain social imaging synchrony; interbrain EEG synchrony; inter-function synchrony; mega interbrain communication; mega interbody communication

Thus, the neurosociological paradigm in the metaverse is extremely new for understanding the function of the social brain in its diversity, including in extraordinary conditions of social interaction in the virtual digital space. It can be assumed that the design of the future metaverse platforms, especially the AI-generated digital platforms, can be conditioned primarily by knowledge about the dynamic features of the functioning of the social neural networks.

## 6 Neurosociology, metaverse and ethics

The evolution of the neurosociological paradigm in the metaverse will be related to the ethics studies. It is well known that social research’s ethical relevance is determined by the progressive codification, institutionalization of ethics research, and the growth of literature in this field (Surmiak and Męcfal, 2024). It is important to emphasize that one of the key factors of ethical issues in social research is the development of new technologies that may entail invasion of participants’ privacy and confidentiality (Mathenjwa et al., 2023). Therefore, it is natural that neuroscience research and new advances in neuroscience raise ethical, social, and legal issues related to humans and their brains, contributing to debates about ethics (Fuchs, 2006; Pickersgill, 2013).

Neuroscience refers to the collection of disciplines concerned with the structure and function of the nervous system and brain (Cacioppo and Decety, 2011). For example, ethical discourse on fMRI is re-emerging, largely influenced by the new interdisciplinary field of neuroethics (Seixas and Ayres Basto, 2008; Shen et al., 2024). The platform providers in the metaverse can collect very large amounts of detailed personal data (records of websites visited, interactions with other users, and the environment). The metaverse can benefit people in the real universe by reducing discrimination, eliminating individual differences, and socialization. However, the metaverse is not exceptional regarding security and privacy concerns (Zhao et al., 2023), the impact of super-realism, and the effects of long-term exposure on the users’ brains (Slater et al., 2020).

The leverage of technologies of the neurosociological paradigm in the metaverse creates new ethical issues that must be explored. The number of metaverse users is still small compared to the number of social media users. Therefore, existing social media regulation could be applied to the metaverse. Still, additional measures will be needed to protect the identity and autonomy of people in the metaverse regarding privacy, identity, property, fraud, abuse, and physical security (Haynes, 2023). An important aspect of maintaining privacy in the metaverse is the evaluation of new/open metaverse applications prior to the stage of anchoring them within the ethical design (Prillard et al., 2024). Finally, AI development in the metaverse will also raise new ethical challenges related to responsible content moderation, ensuring user privacy, and promoting inclusivity (Zhuk, 2024).

In this study, by addressing the ethical issues, we emphasize that while neurosociology has enormous positive potential in digital engagement with the metaverse, significant ethical concerns remain that need to be addressed and discussed.

## 7 Discussion

This study highlights the transformative potential of integrating neurosociology with the metaverse, a fundamentally new scientific and applied area. Future research should explore how these digital environments can be optimized to support diverse social needs including those of neurodivergent populations. In turn, the metaverse social platforms, when integrated with neurosociology, become the virtual environments of new self-managed social interaction for socially solving multidivergent human problems. The neurosociological paradigm in the metaverse developed based on the symbolic interactionism theory (Lee and Joo, 2022), Piaget’s theory of genetic epistemology (Ke and Qiwei, 2020), and our quantization hypothesis will meet the social interests of the wide user audience.

The social aspects of the metaverse from the perspective of the symbolic interactionism theory (Lee and Joo, 2022), show a high degree of identity to the real world, as social interaction in the metaverse is based on the principles of repetitive actions of individuals, through communication in the form of exchange of meaning via language and symbols (Carter and Fuller, 2016).

In the metaverse, the actions of subjects, primarily interpersonal, take place according to the subjective meaning that objects in the real world have for them. In this case, the meanings arise from interactions with others through avatars and virtual objects and are realized in virtual social and cultural contexts. Social interactions with others in the metaverse create meanings and change them in the process of interpretation (Carter and Fuller, 2016). At the same time, however, our study shows that the metaverse as it becomes more widely participatory, demonstrates the phenomenon of limitless expansion and overcoming the limitations of the real world. The objective neurophysiological basis for the realization of the principles of symbolic interactionism in the metaverse as it expands extensively, in our opinion, is the neurosociological paradigm of hyperscanning and interbrain synchrony of the main neural networks of the social brain, which are described in Section 4 in this article.

According to Piaget’s theory of genetic epistemology, subjective interaction with the subject world through actions develops the cognitive structure of social behavior, and recursive processes eventually lead to the achievement of adaptive homeostasis between the subject and the external world (Ke and Qiwei, 2020). According to this theory, the metaverse is the virtual subject world in which the 3D objects or avatars mediate the interaction between subjects in the learning process and forms an adaptive cognitive intersubjects homeostasis. Neurosociological technologies of hyperscanning and interbrain synchrony in social interpersonal interaction/learning in the metaverse objectify the achievement of cognitive adaptive homeostasis at the level of the neural networks of the social brain. The revolutionary nature of the neurosociological paradigm lies in the demand for new technological and social aspects of the metaverse in learning processes, including from the perspective of neurodivergence. In this regard, hyperscanning and interbrain synchrony technologies, in combination with AI, will present a key compartment of the tools for feedback-controlling the content of digital spaces and enhancing them (Figures 3, 4).

Moreover, the evolution of the neurosociological paradigm in the metaverse will lead to the self-evolving metaverse with a highly advanced AI. The neurosociological paradigm in the Metaverse can have applications in the study of healthy function of the social brain (Harris, 2024) as well as dysfunction of the social neural network (DMN, SN, and CEN) based on a pivotal role of AI in detecting disorders by leveraging advanced technologies to analyze vast amounts of data and aid in diagnosis (Jha and Kumar, 2024; Harris, 2024). For example, the demand for the creating the metaverse to promote healthy longevity is very high (Pillay et al., 2024), and the neurosociological paradigm in the metaverse, in our opinion, can represent a unique scientific and applied direction in the health of the social brain.

Cross-domain transitions in the digital spaces are also among the key factors in regulating the main social neural networks and social behavior in the metaverse. Their mechanisms are poorly understood (Zhou et al., 2024), but an unprecedented development of their research can be achieved by integrating the neurosociological paradigm with the metaverse.

The targets of the social brain for the neurosociological paradigm in the metaverse are the neural correlates of the social neural networks DMN, SN, CEN, and reward system at the level of the subcortical network, whose functional significance in the social behavior is well documented (Wirth et al., 2011; Rotge et al., 2015; Krueger et al., 2009; Luo et al., 2018; Elton and Gao, 2014; Seeley, 2019; Uddin, 2014; Bonnelle et al., 2012; Luo et al., 2018; Gabay et al., 2014; Vilares et al., 2011; Bellucci et al., 2016; Krueger et al., 2007; Fareri, 2019; Wu et al., 2016; Montague and Lohrenz, 2007; Tomlin et al., 2013; Xiang et al., 2013; Delgado-Herrera et al., 2021; Zheltyakova et al., 2024; Feng et al., 2021). In this case, the hierarchical interaction between the main social neural networks plays the key role of SN, which determines which network (CEN or DMN), and which nodes of these neural networks are in control at any given time of social behavior (Chand and Dhamala, 2016).

**Figure 5:**
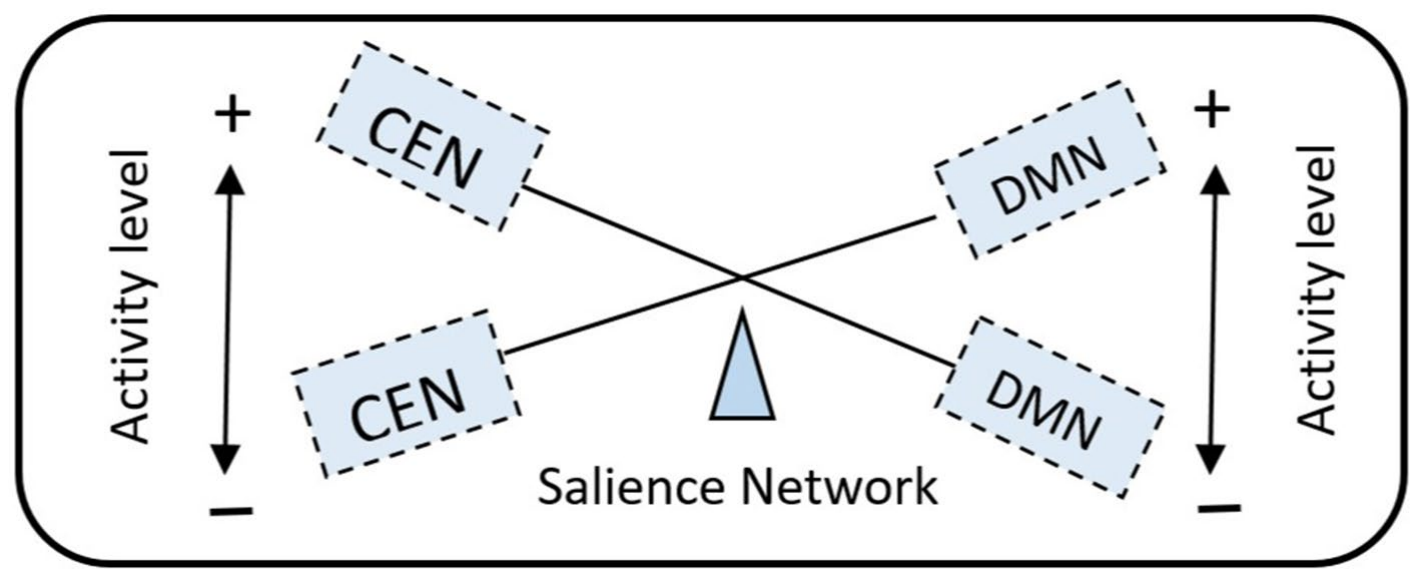
Illustration of the balance between the central executive network (CEN) and default mode network (DMN), regulated by the salience network (SN). This neurophysiological balance is critical for optimizing social behavior and cognitive processes within the metaverse, ensuring users can effectively navigate and interact in virtual environments.

The relationship between the DMN and CEN is a critical balance, and the two networks should not be active simultaneously (Figure 5). Therefore, understanding the dynamic interaction between the large-scale neural networks, primarily DMN, SN, and CEN, when performing complex goal-oriented cognitive tasks in the metaverse defines the strategy for applying the neurosociological paradigm in studying the neurophysiological mechanisms of interaction both within and between DMN, SN, and CEN nodes when making decisions in the process of social interaction in the virtual environment. Moreover, the multistage information processing in the virtual environment increases the information processing ability in the local regions of CEN and SN (Rubinov and Sporns, 2009), which, is the basis of the multistage process of the pioneering neurosociological paradigm in the context of the metaverse. These two digital systems are integrated on the base of the quantization hypothesis of the neurosociological paradigm in the metaverse, the symbolic interactionism theory (Lee and Joo, 2022), and Piaget’s theory of genetic epistemology (Ke and Qiwei, 2020). Based on our quantization hypothesis of the neurosociological paradigm in the metaverse, the directions of the integration can be addressed to improve people’s cognitive activity during social interaction. For example, improving the cognitive processes of attention and/or working memory increases the functional integration of SN and CEN (Luo et al., 2014).

The key targets of integrating the neurosociological paradigm with the metaverse are the neuroplasticity processes in the social brain, which increase with the prolonged use of virtual technology. Thus, the experienced users of virtual systems demonstrate higher levels of processing network information at the level of the social neural networks (Gong et al., 2016). It can be assumed that similar positive results will be achieved by studying the neurodivergence of the social brain from the standpoint of the unique capabilities of the neurosociological paradigm in the metaverse. It should be recognized that the control of the neuroplasticity processes during hyperscanning and interbrain synchrony by biomarkers of social interaction serve as critical feedback mechanisms between the social neural networks and the metaverse environments. We believe that the success of achieving the goals of integration and implementation of the neurosociological paradigm in the metaverse will also be determined by the levels of improvement of the integration of the neurosociological tools with the social virtual platforms and the resources of the real-time monitoring and feedback.

Another key compartment in the hierarchy of social neural networks is the subcortical network (SCN), represented predominantly by the striatum (Feng et al., 2021), which is involved in the hierarchical information processing associated with the social interactions (Alcalá-López et al., 2018; Schurz et al., 2021). The striatum, as a likely input region for heteromodal (affective, cognitive, motor) information, is a heterogeneous structure in terms of intercentral connectivity and functionality that is directly related to the core neural networks of the social brain (Elton and Gao, 2014; Goulden et al., 2014; Greicius et al., 2003; Lee et al., 2012; Vessel et al., 2019), and performs the functions as a central cognitive mechanism of reward response. As a result of understanding the role of the subcortical network of the reward response, it will be possible to implement the affective, cognitive, and motor components of the design of the social virtual environments, which will facilitate the effective adaptation of people from different divergent groups when using the neurosociological paradigm in the metaverse.

Another crucial aspect of realizing the neurosociological paradigm in the metaverse is the inclusion of AI in the digital space. In our opinion, in terms of the new capabilities of AR, VR, and XR glasses for the neurosociological paradigm in the metaverse, these devices should be multisensory for hyperscanning other than electrical (EEG) and/or metabolic (fNIRS) activity of cortical neurons, biomarkers, for example, the levels of emotional states of the VR platforms’ users. Recent findings in this area have been reflected in the recent publications highlighting such features as VR technologies, which have the potential as stress reduction techniques (Ladakis et al., 2024), and personalized VR experiences increasing emotional empathy (Martingano et al., 2021; van Loon et al., 2018). Moreover, the phenomenon of synchrony at the level of executive functions is manifested by physiological responses of the electrodermal activity, peripheral skin temperature (Hanshans et al., 2024), heart rate variability, and pulse variability (Rockstroh et al., 2019; Immanuela et al., 2023). Interestingly, situational factors induce synchrony of HRV in pairs. The stronger HRV synchrony during conflict in pairs predicts greater mood reactivity (Wilson et al., 2018), and it is a biomarker of interpersonal engagement that promotes adaptive learning and effective information sharing during collective decision-making (Sharika et al., 2024).

These facts add new aspects to the multiparameter regulation of subjects’ social behavior in the metaverse and, in general, raise the importance of AI in implementing the VR platforms and improving multisensory VR headsets. They will represent a big data source of the metaverse management by AI, which, in turn, will make it possible to create new integrative indicators of human immersion in social interaction. It can be hypothesized that AI multisensory integration can be a source of the new AI-based feedback integrations in the neurosociological paradigm. A creative example of the realization of this idea can be the work, in which a new integrative index from multiparametric data of large human populations using a deep learning model is the automatic processing and analysis of big data of facial heat maps, metabolic parameters, sleep duration, expression of DNA repair, lipolysis, ATP genes in the blood transcriptome and physical exercises to predict biological age and disease (Yu et al., 2024).

Digital environments can be optimized to support diverse social needs. The brain–machine interface (BMI) facilitates personalized integration of the neurosociological paradigm in the metaverse and appears to be a promising prospect, through which a new channel of direct interaction between the brain, digital platforms, computers, or virtual twins without language and cultural barriers can be created and which will facilitate the development of digital user experience and the adoption of new interpersonal communication channels (Herbert, 2024; Liu Y. et al., 2024; Jia et al., 2024; Kritikos et al., 2023). Optimization to support diverse social needs is the evolution of AR, VR, and XR (Xie et al., 2021; Lee et al., 2021; Dwivedi et al., 2022) and the Internet as an iteration of the metaverse, and not only the social VR platforms within our planet (RecRoom, AltSpaceVR, VRChat, BigScreen, Mozilla Hubs, and Spatial) (Liu and Steed, 2021), but also in the interplanetary space (Vanderdonckt et al., 2024). At the same time, social VR platforms are convenient experimental sites for interdisciplinary neurosociological analysis. Table 2 shows the incredible technological evolution of the neurosociological paradigm in the metaverse, considering the different levels of improvement (Yang et al., 2024a,b) that has occurred in a relatively short period, when the “neurosociological idea” was formulated (TenHouten and Kaplan, 1973; TenHouten, 1997; von Scheve, 2003). This was facilitated by the announcement of the “decade of the brain” initiative which was prompted by neuroscience advances in significant progress in developmental neurobiology, molecular genetics, brain imaging, and computational neuroscience (Tendon, 2000). Since the known social platforms can be classified as precursors of the social metaverse, they allow users to create and manage social interactions in the virtual world. In general view, such a fundamental basis for the realization of the neurosociological paradigm in the metaverse will contribute to the development of a diversity of thought, and social dynamics, as well as solving such a pressing problem as the dual empathy of the neurotypical and neurodivergent people (Norton et al., 2022; Ogawa and Shimada, 2023). Our prospective study highlights the transformative potential of integrating neurosociology with the metaverse. So, on this fundamental basis, future research should explore how these digital environments can be optimized to support diverse social needs, including those of neurodivergent populations.

## 8 Conclusion

In this study we highlight the theoretical and methodological foundations of the neurosociological paradigm in the metaverse and the stages of integrating neurosociology with the metaverse. Currently, the core of the methodology of the neurosociological paradigm in the metaverse is represented by the fNIRS and EEG-based hyperscanning technology and the phenomenon of synchrony of neurophysiological brain biomarkers. The neurobiomarkers of social interaction serve as critical feedback mechanisms between the social neural networks and the metaverse environments during real social interaction. The social neural networks (DMN, SN, CEN and SCN), as well as the social brain as a whole, are the main targets of the research perspective of the transformative potential of neurosociology in the metaverse. Synchronizing biomarkers of the social brain’s activity is a “hallmark” of social interbrain coupling during intergroup communication in the social metaverse. The study cites the digital technologies (AI, BMI and VR headsets) optimized to support diverse social needs. Neurosociology as the science of neural correlates of subjective social interactions, is, in its modern form, a paradigm for redefining the social meaning of the metaverse. The authors suggest that the development of the metaverse will significantly contribute to the technological and conceptual advances in neurosociology. The cutting-edge perspectives of neurosociology will evolve based on the demands of the metaverse rather than those of the real world. The study also discusses essential neurodiversity and ethical aspects of integrating the neurosociological paradigm into the metaverse.

## 9 Limitations

This study is limited to literature studies that examine hyperscanning and interbrain synchrony in VR in the context of the brain-to-brain interaction. The limiting factor in integrating the neurosociological paradigm with the metaverse is the absence of the fundamental technological developments at the real-time monitoring and feedback level. Equally limiting factors are the issues of governance, ethics, security, acceptable behavior, privacy, limited access of the population to the metaverse digital infrastructure, and unregulated social norms within the virtual environments. The crucial factor in the limitations of the neurosociological paradigm in the metaverse is subjective. When an individual is immersed in the virtual environment, risks are associated with the impact of rich and varied visual and auditory sensory experiences on the emotional well-being and physical sensations. It initiates anxiety, nausea and eye fatigue in users. Elderly adults exposed to VR experience an even greater range of limitations in the form of lack of skills in using interface/design applications, lack/low digital literacy, low awareness of cyber safety, and limited access to digital devices and the Internet. It should be recognized that fNIRS, EEG, and BMI headsets, as well as probable technological improvements in the field of application of the neurosociological paradigm in the form of multisensory technologies, will require developers of new solutions, since the above subjective and technical limitations are the experience of the previous development of the VR platforms and neurophysiological equipment. Limiting factors related to a general vision of the metaverse are discussed in the scientific debates (Cerasa et al., 2022; Dwivedi et al., 2022; Girginova, 2024).

## Data Availability

The original contributions presented in the study are included in the article/supplementary material; further inquiries can be directed to the corresponding author(s).
